# Effects of Virtual Reality on Postoperative Pain Management Following Minimally Invasive Gynecologic Surgery: Randomized Controlled Trial

**DOI:** 10.2196/92442

**Published:** 2026-07-02

**Authors:** Qiyue Hu, Liyuan Chen, Yuye Zhu, Tingting Xiang, Xia Liang, Guiyun Wu, Miao Ding, Tengfei Long

**Affiliations:** 1 Sun Yat-sen Memorial Hospital of Sun Yat-sen University Guangzhou City, Guangdong China; 2 The First People's Hospital of Tianmen City in Hubei province Tianmen, Hubei Province China

**Keywords:** virtual reality, VR, minimally invasive surgery, gynecology, postoperative pain, anxiety, randomized controlled trial

## Abstract

**Background:**

Postoperative pain and anxiety remain common concerns after minimally invasive gynecologic surgery despite advances in surgical techniques and analgesic strategies. Virtual reality (VR) has been investigated as a potential nonpharmacological intervention for pain management; however, evidence in gynecologic postoperative settings is limited.

**Objective:**

This study aims to evaluate the efficacy and safety of VR technology compared with standard postoperative analgesia for pain and anxiety management in patients undergoing minimally invasive gynecologic surgery.

**Methods:**

This randomized controlled trial was conducted at Sun Yat-sen Memorial Hospital of Sun Yat-sen University in China. A total of 131 patients undergoing laparoscopy or combined hysteroscopy for benign gynecologic diseases were randomly assigned in a 1:1 ratio to either a VR group (n=68) or a control group (n=63). All patients received a standardized general anesthesia protocol intraoperatively. The control group received conventional analgesic therapy after surgery, and the VR group received a 20-minute VR intervention 6 hours postoperatively. The pain and anxiety levels were evaluated using a visual analog scale at 6 and 7 hours postoperatively. The primary outcome was the change in pain scores between 6 and 7 hours. Secondary outcomes included maximum pain score, anxiety score changes, length of hospital stay, hospitalization costs, and occurrence of adverse events. Analyses were performed according to the intention-to-treat principle.

**Results:**

There was no statistically significant difference in the primary outcome between the VR and control groups (mean difference 0.169, 95% CI −0.271 to 0.608; *P*=.45). Similarly, no significant differences were observed in the maximum pain score (mean difference 0.839, 95% CI −0.101 to 1.779; *P*=.08), and no improvement was observed in the anxiety score (mean difference 0.042, 95% CI −0.365 to 0.449; *P*=.84). No significant differences were found in length of hospital stay, hospitalization costs, or incidence of adverse events, including dizziness, nausea, and vomiting (all *P*>.05).

**Conclusions:**

A single 20-minute VR intervention did not provide additional analgesic or anxiolytic benefit compared with standard postoperative care after minimally invasive gynecologic surgery. VR was well tolerated, and its role in postoperative recovery requires further investigation.

**Trial Registration:**

Chinese Clinical Trial Registry ChiCTR2400091244; https://tinyurl.com/4b92a9td

## Introduction

Minimally invasive gynecologic surgery has become the mainstream treatment for benign gynecologic diseases, with advantages of reduced surgical trauma, faster recovery, and shorter hospital stay compared with open surgery [1]. However, postoperative pain (including abdominal distension, shoulder pain, and incision pain) and anxiety are still common clinical problems, which delay early mobilization, increase complication risk, and reduce patient quality of life [2,3]. Within the enhanced recovery after surgery (ERAS) framework, effective postoperative pain and anxiety management is essential. Although traditional analgesic strategies (such as nonsteroidal anti-inflammatory drugs, opioid analgesics, and analgesic pumps) are widely used, they are limited by side effects (eg, nausea, vomiting, intestinal obstruction, and drug dependence); high cost; and individual ineffectiveness [4]. Therefore, there is an urgent need to explore safe, nonpharmacological, and low-cost adjuvant interventions in clinical practice.

Virtual reality (VR) provides a 3D immersive experience and has been reported to reduce pain and anxiety by diverting attention in various acute pain scenarios [5,6]. A recent meta-analysis including 92 randomized controlled trials and 7133 participants showed that immersive VR can effectively reduce pain perception during invasive medical operations [7]. Potential benefits of VR have also been reported in chronic pain management, physical rehabilitation, mental health treatment, oral surgery, and pediatric burn care [8-12]. However, high-quality evidence of VR for postoperative pain and anxiety after minimally invasive gynecologic surgery is still insufficient [13]. Previous studies evaluating VR interventions in perioperative settings have been limited by small sample sizes, heterogeneous intervention protocols, and inconsistent outcome measures [14]. The optimal timing, duration, and frequency of VR application in this population remain unclear.

To fill this research gap, we conducted this randomized controlled trial. The primary research purpose is to evaluate whether a single 20-minute VR intervention at 6 hours postoperatively can further reduce pain and anxiety compared with standard analgesia in patients undergoing minimally invasive gynecologic surgery. The secondary purposes are to assess the effects of VR on hospital stay, hospitalization cost, and adverse events and to assess the safety and feasibility of VR in clinical practice. We hypothesized that VR might provide additional nonpharmacological analgesic and anxiolytic benefits without increasing medical burden.

## Methods

### Research Objectives

Eligible patients were consecutively screened and recruited from the Department of Obstetrics and Gynecology at Sun Yat-sen Memorial Hospital of Sun Yat-sen University between April 2021 and April 2022. All patients scheduled for laparoscopic or combined hysteroscopic surgery for benign gynecologic conditions were assessed for eligibility according to predefined inclusion and exclusion criteria (Textbox 1). This consecutive recruitment approach helped reduce selection bias and improve representativeness during the study period.

**Eligibility criteria.**

**Inclusion criteria**

- Nonpregnant women aged 18 to 70 years, admitted for benign gynecologic diseases and scheduled to undergo laparoscopy or combined hysteroscopy
- Willingness to participate in the study
- Ability to read and independently complete questionnaires

**Exclusion criteria**

- Had suspected malignancy, anatomical abnormalities, severe comorbidities, sensory impairments, unwillingness to participate, or a history of more than 3 prior abdominal surgeries

**Exit criteria**

- Serious complications during the study
- The operation was canceled for any reason
- Severe intolerance to pain
- The patient requested to withdraw from the study

### Ethical Considerations

This study was approved by the ethics committee of Sun Yat-sen Memorial Hospital, Sun Yat-sen University (SYSKY-2023-983-02). All participants provided written informed consent prior to enrollment. All patient data were anonymized and deidentified to strictly protect personal privacy and information confidentiality. No financial compensation was provided to the participants in this study.

### Trial Registration

This randomized controlled trial was retrospectively registered. The research team was not fully aware of the International Committee of Medical Journal Editors prospective registration policy at the time of study initiation, and participant enrollment began before formal trial registration was completed. Retrospective registration was subsequently completed in compliance with the International Committee of Medical Journal Editors guidelines and JMIR Publications requirements (Chinese Clinical Trial Registry ChiCTR2400091244).

### Randomization and Blinding

Participants were randomly assigned to either the VR or control group using a computer-generated random sequence prepared by an independent statistician. Sequentially numbered, sealed, opaque envelopes were used to implement distribution and concealment. Only after the research coordinator obtained the informed consent of the patient can the corresponding envelope be opened. Due to the nature of VR therapy, patients and health care providers were aware of the intervention plan, but outcome assessors who collected postoperative pain and anxiety data were blinded to group allocation. This approach reduced the risk of assessment bias and improved the methodological rigor of the trial.

### Research Procedure

The study used an exploratory study design. We aimed to evaluate the potential effect of VR technology on postoperative pain and anxiety in patients undergoing minimally invasive gynecologic surgery. Participants were randomized into VR (n=68) and control (n=63) groups by sealed envelopes. The control group received conventional analgesic drugs for pain relief, and the VR group received only VR technology without any analgesic drugs. Patients in the VR group received a 20-minute VR session in the gynecology ward 6 hours postoperatively, using portable standalone VR glasses with 3D dynamic content. Demographic and clinical baseline data of the participants were collected at randomization, including age, pregnancy, menopausal status, number of previous operations, and surgical methods. Before surgery, a baseline psychological state assessment was conducted. We used standardized scales that were validated for reliability and validity, including the Self-Rating Anxiety Scale (SAS), the Self-Rating Depression Scale (SDS), and the Pain Catastrophizing Scale (PCS). All participants completed these assessments prior to randomization to ensure comparability between groups. In this study, all participants received interventions according to the study protocol. The VR intervention was delivered by trained health care providers who were responsible for setting up and monitoring the VR sessions.

All patients in both groups were treated with a standardized general anesthesia regimen. Anesthesia was induced with intravenous sufentanil (0.3 μg/kg), cisatracurium (0.2 mg/kg), and propofol (2-3 mg/kg), followed by tracheal intubation and mechanical ventilation. Anesthesia was maintained intraoperatively using sevoflurane at 0.7 to 1.0 minimum alveolar concentration, with the bispectral index maintained between 40 and 60. At the end of surgery, sevoflurane was discontinued, and patients were transferred to the postanesthesia care unit. During postoperative resuscitation, intravenous sufentanil (0.1-0.2 μg/kg) was administered as needed to maintain a visual analog scale (VAS) pain score below 2. After recovery from anesthesia, the patients were transferred to the general ward after awakening from anesthesia.

The control group received a routine multimodal analgesic regimen consisting of nonsteroidal anti-inflammatory drugs (eg, celecoxib 100 mg every 12 hours), transcutaneous electrical nerve stimulation, and tramadol (30-60 mg intravenously or intramuscularly) as needed.

Patients in the VR group received a 20-minute VR intervention initiated at 6 hours postoperatively, without the use of routine postoperative analgesic medications. The VR intervention was initiated at 6 hours postoperatively, at which time the main effects of intraoperative and immediate postoperative analgesics were expected to have largely diminished, but some residual effects may still exist.

To promote patient safety and ethical compliance, the researchers closely monitored the patients in the VR group. If the patient has severe pain, the corresponding analgesic regimen will be given immediately according to clinical need. Such patients were withdrawn from the intervention protocol to reduce potential confounding.

### Outcomes

The primary outcome was the improvement in VAS score for pain at 7 and 6 hours postoperatively. The VAS scores are divided into 0 to 10 points on a number scale. A score of 0 indicates no pain or anxiety, and a score of 10 indicates unbearable severe pain or anxiety. Secondary outcomes included the maximum pain score and improvement in anxiety levels assessed using the VAS for anxiety. Total length of stay, total hospitalization cost, and adverse events such as dizziness, nausea, and vomiting were also recorded in each group.

### Sample Size Calculation

The sample size was calculated based on the expected difference in postoperative pain scores (VAS) between the VR and control groups, using data from a previous clinical study by Steinkraus et al [15]. Assuming a 2-sided significance level of α=.05, a power of 80%, and an expected mean difference in VAS scores of 0.33 with equal variances between groups, a minimum of 32 participants per group was required. To account for an anticipated 20% dropout rate, we planned to recruit at least 39 participants per group, for a total of 78 patients. This calculation was intended to provide adequate statistical power to detect clinically meaningful differences in postoperative pain between the intervention and control groups.

### Statistical Processing

The primary outcome was analyzed using a generalized linear model with an identity link function, including the treatment group (VR vs control) as a fixed effect and the baseline VAS score as a covariate. Adjusted mean differences with 95% CIs were estimated from the model.

Secondary continuous outcomes were analyzed using similar generalized linear model approaches. For outcomes with a single postbaseline measurement, the treatment group was included as the sole predictor. Mean differences with 95% CIs were reported. No significant deviations from normality were observed, supporting the use of mean (SD) for data presentation. Normality of continuous outcomes was assessed visually using Q-Q plots and histograms (Figures S1-S5 in Multimedia Appendix 1), which supported the use of parametric analyses.

The initial analysis was not corrected for confounders. Additionally, covariate-adjusted analyses were conducted for all outcomes, adjusting for age, type of surgery, number of previous surgeries, and gravidity. No formal within-group inferential analyses were prespecified. All analyses were performed according to the intention-to-treat principle, including all eligible randomized patients. No missing data were observed for primary or secondary outcomes. All statistical tests were 2-sided, and a *P*<.05 was considered statistically significant. Statistical analyses were performed using SPSS (version 26.0; IBM Corp) and Prism (version 9.5; GraphPad).

## Results

### Patient Characteristics

**Figure 1:**
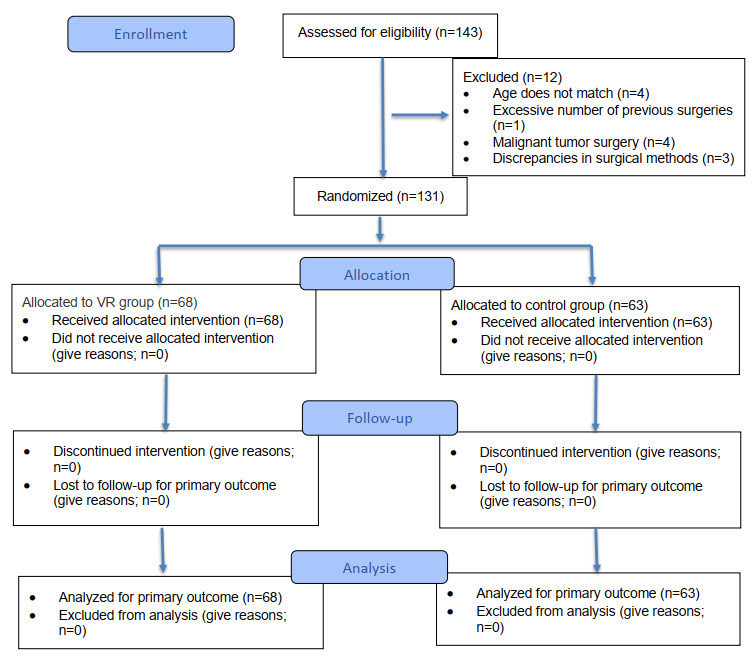
Flow chart of participants inclusion and exclusion.

A total of 143 patients were initially randomized; 12 patients were excluded for not meeting the inclusion criteria. All remaining patients completed the questionnaires during hospitalization, with no loss to follow-up. Therefore, 68 patients in the VR group and 63 in the control group were included in the final analysis (Figure 1). Baseline characteristics, including age, parity, menopausal status, number of previous operations, and type of surgery, were comparable between the 2 groups (all *P*>.05). Preoperative psychological assessments, including anxiety (SAS), depression (SDS), and PCS, also showed no statistically significant differences between groups (all *P*>.05), suggesting baseline comparability between groups (Table 1).

**Table 1:** Baseline demographic and clinical characteristics of participants in the VR^a^ group and the control group (N=131).

		Group	
		VR group (n=68)	Control group (n=63)
Age (years), mean (SD)		37.46 (10.276)	37.10 (10.038)
**Parity, n (%)**			
	Nullipara	18 (26.5)	26 (41.3)
	Multipara	50 (73.5)	37 (58.7)
**Menopausal state, n (%)**			
	Premenopause	61 (89.7)	59 (93.7)
	Menopause	7 (10.3)	4 (6.3)
Number of previous operations, mean (SD)		1.56 (1.343)	1.13 (1.338)
**Mode of operation, n (%)**			
	Laparoscope	35 (51.5)	29 (46)
	Laparoscopy and hysteroscopy	33 (48.5)	34 (54)
VAS score at 6 hours, mean (SD)		1.62 (1.901)	1.92 (2.491)
Anxiety score at 6 hours, mean (SD)		2.56 (2.018)	2.49 (2.758)
Dizziness and nausea score, mean (SD)		1.62 (1.901)	1.92 (2.491)
Vomiting score, mean (SD)		0.35 (0.842)	0.21 (0.600)
SAS^b^, mean (SD)		37.61 (5.712)	36.37 (6.381)
SDS^c^, mean (SD)		46.29 (6.800)	45.51 (7.381)
PCS^d^, mean (SD)		31.63 (8.921)	29.17 (10.753)

^a^VR: virtual reality.

^b^SAS: Self-Rating Anxiety Scale.

^c^SDS: Self-Rating Depression Scale.

^d^PCS: Pain Catastrophizing Scale.

### Primary Outcome

The primary outcome, defined as the improvement in the VAS score between 6 and 7 hours postoperatively, did not differ significantly between the VR and control groups. In the unadjusted analysis, the mean difference was 0.169 (95% CI −0.271 to 0.608; *P*=.45). After adjustment for prespecified covariates (age, type of surgery, number of previous surgeries, and gravidity), the result remained nonsignificant (mean difference 0.136, 95% CI −0.300 to 0.573; *P*=.54; Table 2).

**Table 2:** Between-group comparisons of postoperative pain and anxiety scores. Adjusted mean differences with 95% CIs were derived from generalized linear models, with baseline VAS score included as a covariate.

		Group, mean (SD)			Unadjusted			Adjusted	
		VR^a^ group	Control group	Mean difference (95% CI)		*P* value	Mean difference (95% CI)		*P* value
**Primary outcome**									
	Improvement in VAS^b^ score (6-7 hours)	0.93 (1.083)	0.71 (1.549)	0.169 (-0.271 to 0.608)		.45	0.136 (-0.300 to 0.573)		.54
**Secondary outcomes**									
	Maximum pain score	4.50 (2.175)	3.66 (3.188)	0.839 (-0.101 to 1.779)		.08	0.801 (-0.149 to 1.750)		.10
	Improvement in anxiety degree (6-7 hours)	0.66 (0.908)	0.60 (1.642)	0.042 (-0.365 to 0.449)		.84	0.043 (-0.368 to 0.454)		.84
	Total length of stay (days)	6.19 (1.739)	6.51 (1.900)	−0.317 (-0.946 to 0.312)		.32	−0.306 (-0.904 to 0.292)		.31
	Total hospitalization costs, CNY^c^	24,547.41 (8189.616)	24,439.98 (5430.464)	107.426 (-2314.644 to 2529.495)		.93	−46.541 (-2093.137 to 2000.055)		.96
	Improvement in the dizziness and nausea score	0.54 (1.177)	0.54 (1.554)	−0.303 (-1.066 to 0.460)		.43	−0.289 (-1.059 to 0.481)		.46
	Improvement in the vomiting score	0.03 (0.690)	−0.10 (0.640)	0.147 (-0.108 to 0.401)		.26	0.146 (-0.112 to 0.405)		.27

^a^VR: virtual reality.

^b^VAS: visual analog scale.

^c^CNY 1=US $0.15 as of June 5, 2026.

### Secondary Outcomes

No statistically significant differences were observed between the two groups for any secondary continuous outcomes. Maximum pain score showed a nonsignificant trend toward higher values in the VR group (unadjusted mean difference 0.839, 95% CI −0.101 to 1.779; *P*=.08; adjusted *P*=.10).

Improvement in anxiety scores (6-7 hours) was comparable between groups (unadjusted mean difference 0.042, 95% CI −0.365 to 0.449; *P*=.84; adjusted *P*=.84).

Total length of hospital stay did not differ significantly (unadjusted mean difference −0.317 days, 95% CI −0.946 to 0.312; *P*=.32; adjusted *P*=.31).

Total hospitalization costs were also similar between groups (unadjusted mean difference 107.426 CNY, 95% CI −2314.644 to 2529.495 [CNY 1=US $0.15 as of June 5, 2026]; *P*=.93; adjusted *P*=.96; Table 2).

### Postoperative Symptoms

Postoperative symptom improvements, including dizziness, nausea, and vomiting, were analyzed as continuous variables. No statistically significant differences were found between groups in the improvement in dizziness and nausea scores: mean difference −0.303 (95% CI −1.066 to 0.460; *P*=.433; adjusted *P*=.46)

Improvement in vomiting score: mean difference 0.147 (95% CI −0.108 to 0.401; *P*=.26; adjusted *P*=.27)

These findings suggest that VR intervention was not associated with significant improvements in postoperative symptom recovery compared with the control group (Table 2).

### Summary of Findings

Overall, neither the primary outcome nor any secondary continuous outcomes showed statistically significant differences between the VR and control groups in either unadjusted or adjusted analyses.

## Discussion

### Principal Findings

This randomized controlled trial enrolled 131 patients undergoing minimally invasive gynecologic surgery to evaluate the efficacy and safety of VR for postoperative pain and anxiety management. The primary outcome—change in VAS score between 6 and 7 hours postoperatively—showed no significant difference between the VR group and the control group (mean difference 0.169, 95% CI −0.271 to 0.608; *P*=.45). For secondary outcomes, maximum pain score, anxiety improvement, length of hospital stay, hospitalization cost, and incidence of dizziness, nausea, and vomiting were also comparable between groups (all *P*>.05). The absence of between-group differences suggests that a single postoperative VR session may provide limited additional benefit beyond routine perioperative care in this population. No increase in adverse events or hospitalization costs was observed in the VR group.

### Interpretation of Results

VR has been proposed to reduce pain perception by diverting attention away from unpleasant stimuli [16]. Previous studies have reported reduced pain and anxiety during short procedural interventions using VR. A clinical study demonstrated that the use of VR during pediatric venipuncture effectively reduces pain and anxiety in children [17]. VR has been associated with reductions in intraoperative pain and anxiety among children undergoing circumcision and nasal endoscopy [18,19]. However, this study did not identify superior analgesic and anxiolytic effects of VR relative to conventional drug therapy. Gray et al [20] found that VR significantly reduced anxiety in pediatric and adult nasal procedures, respectively, but pain improvement was inconsistent.

With regard to the surgical type, a systematic review by Levit et al [21] showed that VR may reduce pain and anxiety in minor surgeries, but the evidence in medium to large surgeries is limited. Our study included laparoscopic and combined hysteroscopic laparoscopic surgeries. The relatively low postoperative pain scores observed in this cohort may have limited the ability to detect additional benefits from VR. Patients enrolled in this study presented moderate preoperative baseline scores on both the SAS and the PCS. Baseline anxiety and catastrophizing scores were relatively low, which may have reduced the observable effect size of the intervention.

In terms of intervention protocol, Chan et al [22] conducted a randomized controlled trial in gynecologic surgery and found that 3 sessions of VR (10-20 minutes each) within 24 hours postoperatively significantly reduced pain and anxiety scores at 24, 48, and 72 hours. In contrast, our study adopted only one 20-minute VR session at 6 hours, with a shorter duration and lower frequency. The longer intervention schedule used in previous studies may partly explain the different findings across studies [23]. The single-session design used in this study may have limited the sustained effect of VR on postoperative symptoms. In subsequent clinical applications and experimental designs, VR can be adopted repeatedly within a certain period after surgery to observe its therapeutic effects on pain and anxiety.

Differences in VR content and device characteristics across studies may also contribute to heterogeneity in reported outcomes. Dreesmann et al [24] suggested that immersion quality and VR content design may influence the therapeutic efficacy of VR interventions. Khadra et al [25] adopted projection-based VR with a wide-field screen, which was associated with greater immersion and improved pain relief outcomes in pediatric burn care. In comparison, the head-mounted VR devices used in this study provide a relatively confined field of view, but the impact of device type on clinical outcomes remains unclear and warrants further study.

Furthermore, this intervention did not increase the incidence of adverse events such as dizziness, nausea, and vomiting nor did it raise hospitalization costs. This was consistent with Andersen et al [26], suggesting that VR interventions are generally well tolerated and safe in clinical settings. Additional studies are needed to clarify whether VR provides clinically meaningful benefits in postoperative gynecologic care.

### Strengths and Limitations

This study has several strengths. It is one of the few randomized controlled trials evaluating VR technology for pain management after gynecologic surgery. It also used a randomized design with intention-to-treat analysis and standardized perioperative management to enhance internal validity. In addition, validated psychological assessment tools (SAS, SDS, and PCS) were used for a comprehensive baseline evaluation. Although we did not observe that VR technology had better analgesic and anxiolytic effects than conventional analgesic regimens, the findings supported the safety and feasibility of VR intervention in postoperative gynecologic care settings. Compared with previous VR-related studies, the relatively larger sample size compared with previous studies may reduce random error [27].

There are also several limitations that need to be addressed in this study. This study was a single-center study and may limit the extrapolation of the findings. The study did not collect detailed information about the participants’ previous history of mental illness and related medications, leading to potential confounding factors. Furthermore, relatively low postoperative pain and anxiety levels, a short intervention duration, and a modest sample size may have reduced the ability to detect substantial differences. Further multicenter studies with longer follow-up and standardized VR protocols are warranted.

### Conclusions

In conclusion, a single 20-minute VR intervention did not provide additional analgesic or anxiolytic benefit compared with standard postoperative care after minimally invasive gynecologic surgery. Further studies are needed to determine whether different intervention protocols or patient populations may benefit from VR-based perioperative support.

## Appendix

Multimedia Appendix 1 Q-Q plots of residual distributions for continuous outcomes.

Multimedia Appendix 2 CONSORT-eHEALTH checklist (V 1.6.1).

## Abbreviations

PCS: Pain Catastrophizing Scale
ERAS: enhanced recovery after surgery
SAS: Self-Rating Anxiety Scale
SDS: Self-Rating Depression Scale
VAS: visual analog scale
VR: virtual reality

## Data Availability

All data are available upon reasonable request to the corresponding author.
