# Supplementary material for: Effects of Virtual Reality on Postoperative Pain Management Following Minimally Invasive Gynecologic Surgery: Randomized Controlled Trial
Source: JMIR Form Res. 2026 Jul 2;10:e92442. doi: 10.2196/92442 (PMC13376855; doi:10.2196/92442)
Supplement: Multimedia Appendix 1 [file formative_v10i1e92442_app1.docx]

**Supplementary Figure S1-5. Q–Q plots of residual distributions for continuous outcomes.**


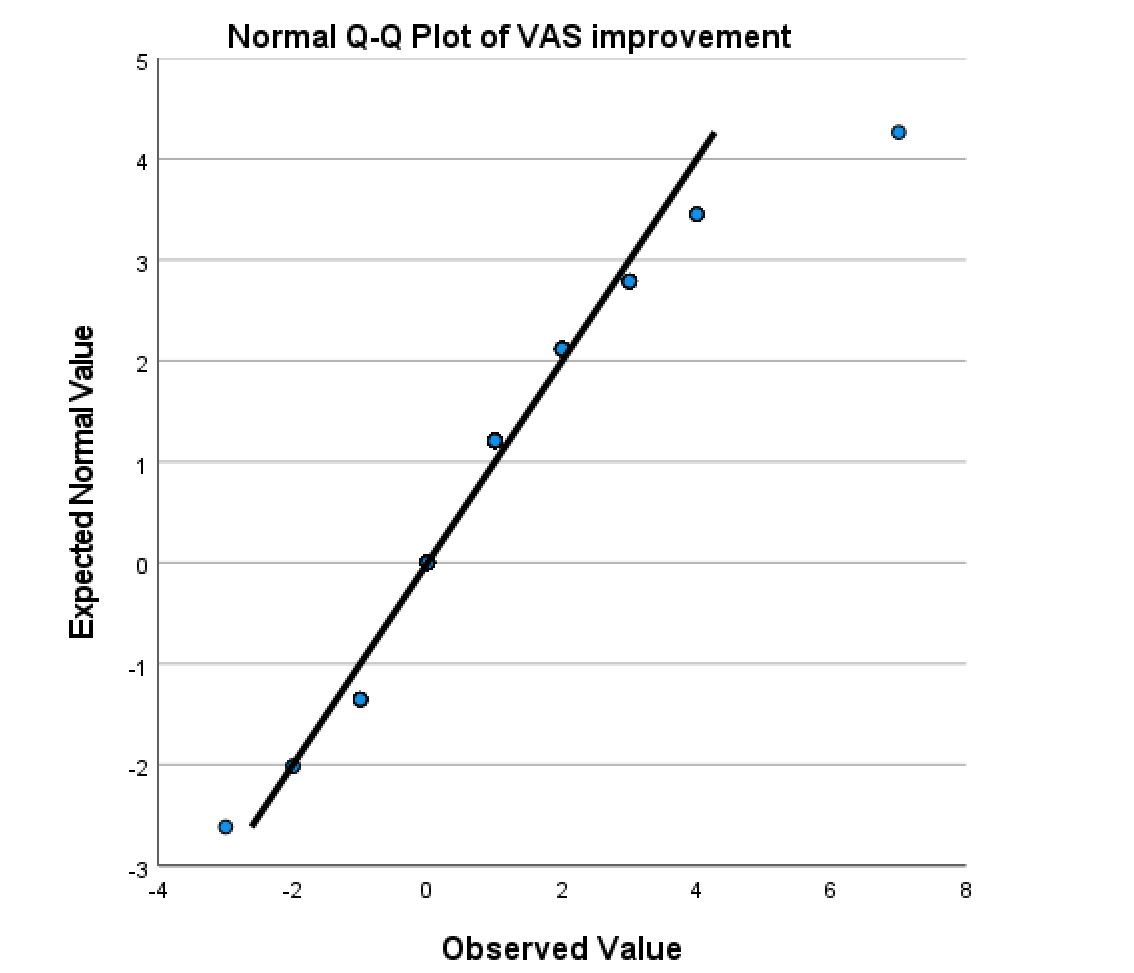


Supplementary Figure S1. Q-Q plot of the difference in pain scores


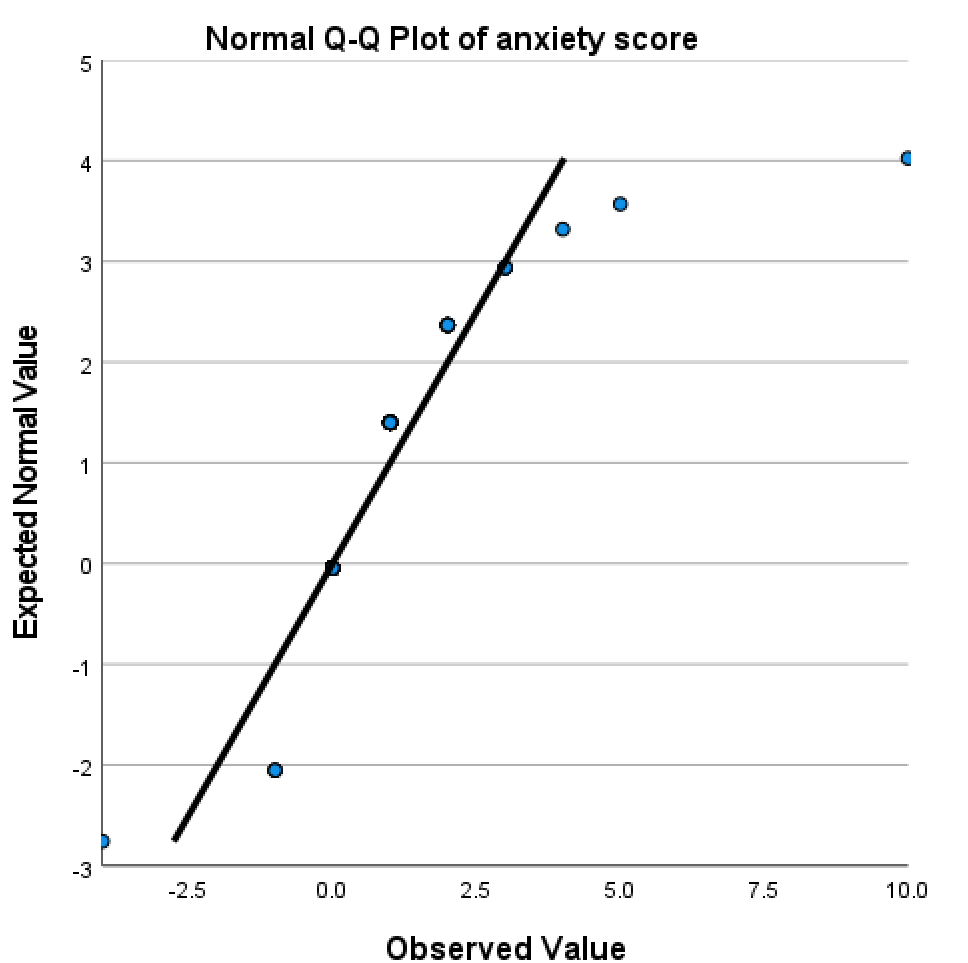


Supplementary Figure S2. Q-Q plot of the difference in anxiety scores


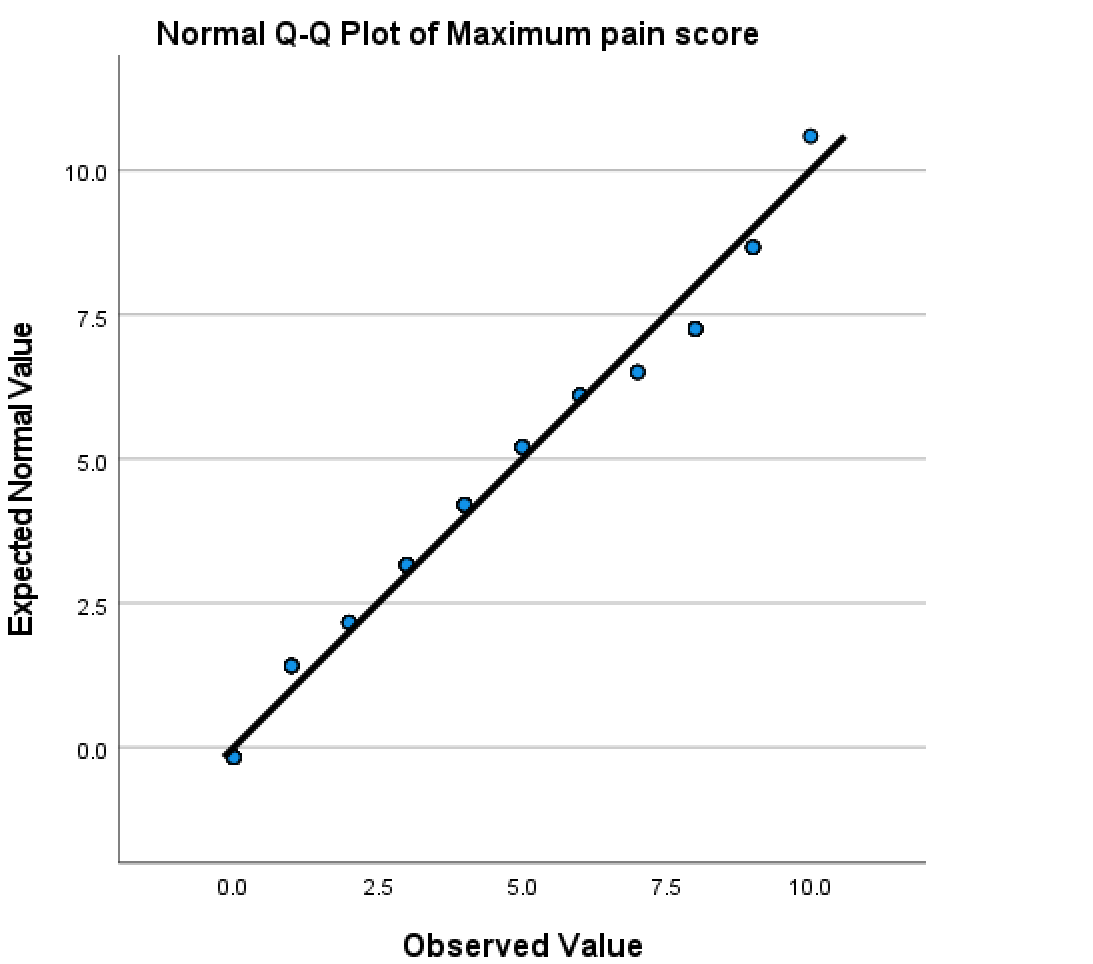


Supplementary Figure S3. Q-Q plot of the difference in maximum pain score


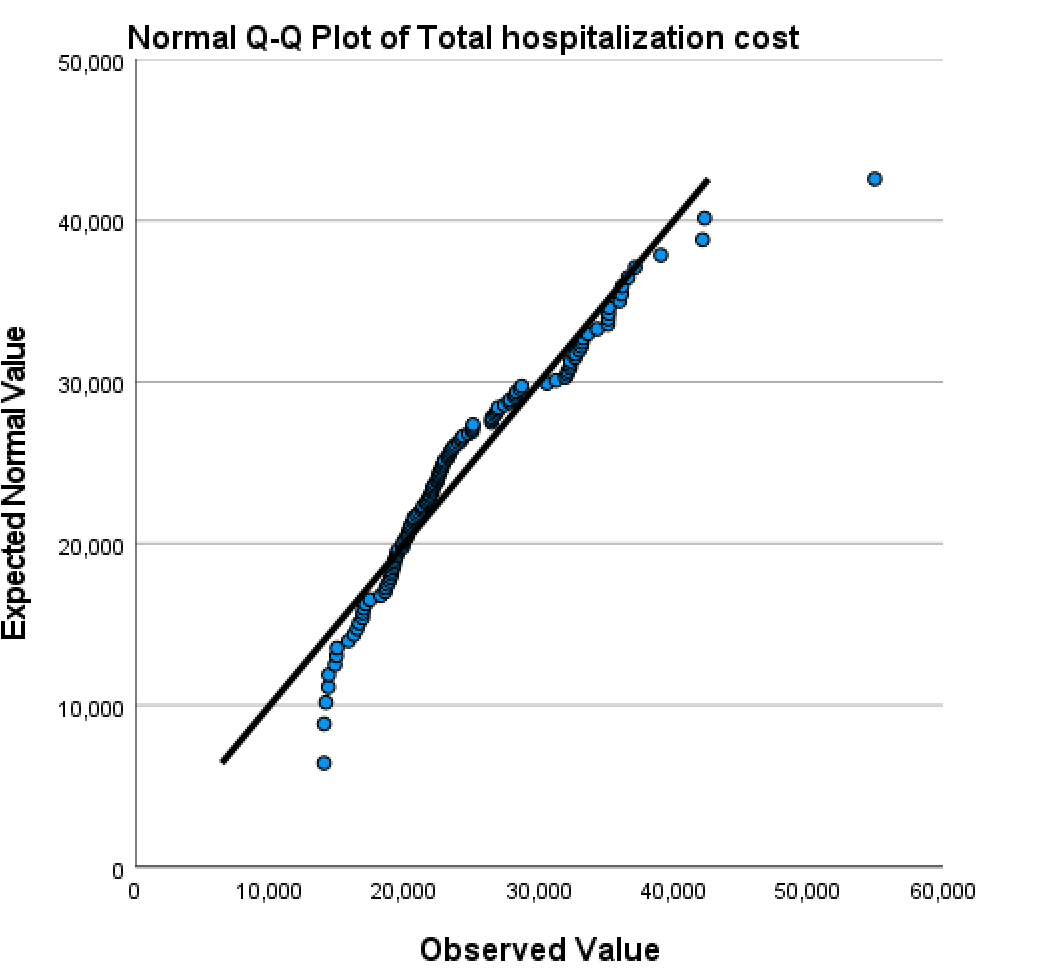


Supplementary Figure S4. Q-Q plot of the difference in total hospitalization cost


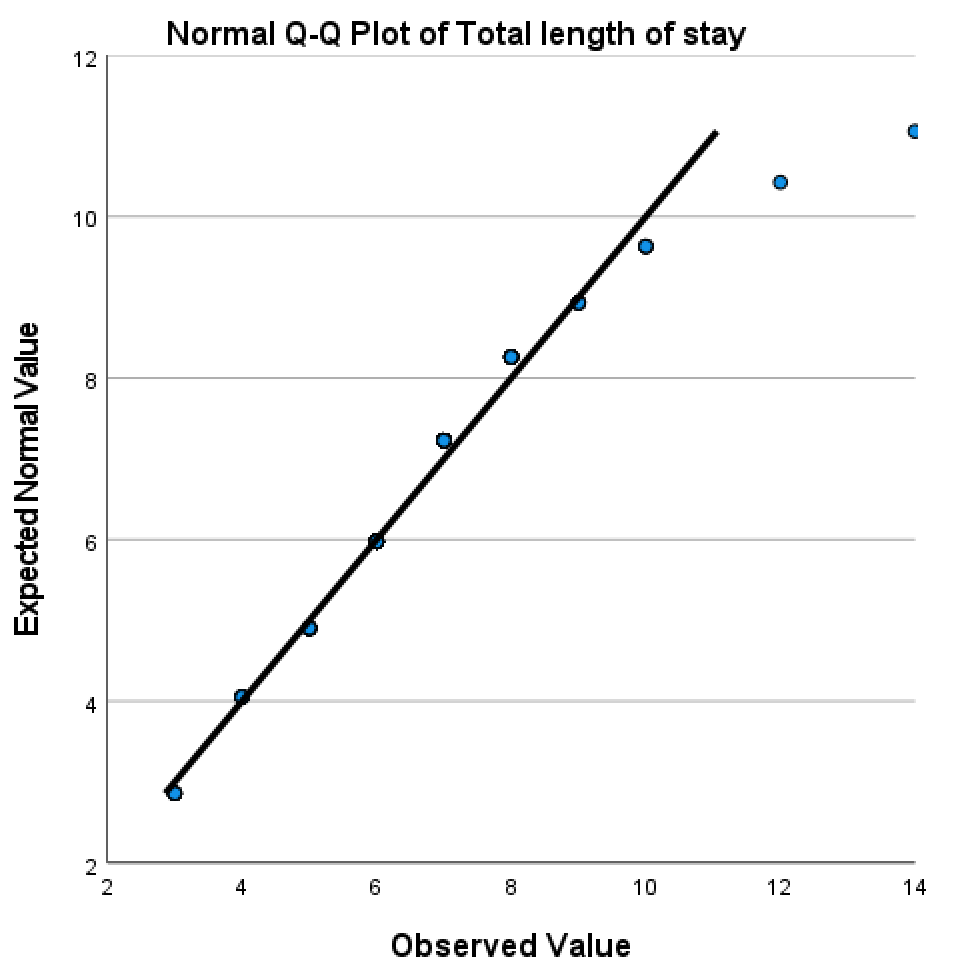


Supplementary Figure S5. Q-Q plot of the difference in total length of hospital stay
