# Supplementary material for: Effects of Virtual Reality on Postoperative Pain Management Following Minimally Invasive Gynecologic Surgery: Randomized Controlled Trial
Source: JMIR Form Res. 2026 Jul 2;10:e92442. doi: 10.2196/92442 (PMC13376855; doi:10.2196/92442)
Supplement: Multimedia Appendix 2 [file formative_v10i1e92442_app2.pdf]

CONSORT-EHEALTH (V 1.6.1) - Submission/Publication Form

The CONSORT-EHEALTH checklist is intended for authors of randomized trials evaluating web-based and Internet-based applications/interventions, including mobile interventions, electronic games (incl multiplayer games), social media, certain telehealth applications, and other interactive and/or networked electronic applications. Some of the items (e.g. all subitems under item 5 - description of the intervention) may also be applicable for other study designs.

The goal of the CONSORT EHEALTH checklist and guideline is to be
a) a guide for reporting for authors of RCTs,
b) to form a basis for appraisal of an ehealth trial (in terms of validity)

CONSORT-EHEALTH items/subitems are MANDATORY reporting items for studies published in the Journal of Medical Internet Research and other journals / scientific societies endorsing the checklist.

Items numbered 1., 2., 3., 4a., 4b etc are original CONSORT or CONSORT-NPT (non-pharmacologic treatment) items.
Items with Roman numerals (i., ii, iii, iv etc.) are CONSORT-EHEALTH extensions/clarifications.

As the CONSORT-EHEALTH checklist is still considered in a formative stage, we would ask that you also RATE ON A SCALE OF 1-5 how important/useful you feel each item is FOR THE PURPOSE OF THE CHECKLIST and reporting guideline (optional).

Mandatory reporting items are marked with a red *.
In the textboxes, either copy & paste the relevant sections from your manuscript into this form - please include any quotes from your manuscript in QUOTATION MARKS,
or answer directly by providing additional information not in the manuscript, or elaborating on why the item was not relevant for this study.

YOUR ANSWERS WILL BE PUBLISHED AS A SUPPLEMENTARY FILE TO YOUR PUBLICATION IN JMIR AND ARE CONSIDERED PART OF YOUR PUBLICATION (IF ACCEPTED).
Please fill in these questions diligently. Information will not be copyedited, so please use proper spelling and grammar, use correct capitalization, and avoid abbreviations.

DO NOT FORGET TO SAVE AS PDF _AND_ CLICK THE SUBMIT BUTTON SO YOUR ANSWERS ARE IN OUR DATABASE !!!

Citation Suggestion (if you append the pdf as Appendix we suggest to cite this paper in the caption):
Eysenbach G, CONSORT-EHEALTH Group
CONSORT-EHEALTH: Improving and Standardizing Evaluation Reports of Web-based and Mobile Health Interventions
J Med Internet Res 2011;13(4):e126

URL: http://www.jmir.org/2011/4/e126/
doi: 10.2196/jmir.1923
PMID: 22209829

登录 Google 即可保存进度。了解详情

* 表示必填

Your name *

First Last

Qiyue Hu

Primary Affiliation (short), City, Country *

University of Toronto, Toronto, Canada

Sun Yat-sen Memorial Hospital of Sun Yat-sen

Your e-mail address *

abc@gmail.com

huqy27@mail.sysu.edu.cn

Title of your manuscript *

Provide the (draft) title of your manuscript.

Effects of Virtual Reality on Postoperative Pain Following Minimally Invasive Gynecologic Surgery: A Randomized Controlled Trial

你的应用/软件/干预名称 *

如果有短名和长名/备用名，先写短名，括号内加长名。

Virtual reality (VR)

评估版本（如有）

例如：“V1”、“发布 2017-03-01”、“版本 2.0.27913”

您的回答

语言 *

干预/申请用什么语言？如果有多种语言，请用逗号分隔（例如“英语、法语”）

Chinese

您的干预网站或应用网址

例如，在应用商店（iTunes、Google Play）中直接指向移动应用的链接，或网站的网址。如果介入是DVD或硬件，你也可以链接到亚马逊页面。

您的回答

图片/截图的网址（可选）

您的回答

交通便利性 *

终端用户现在能访问干预措施吗?

- [x] 免费开放
- [ ] 仅限特殊用户组访问，不公开
- [ ] 访问权限对所有人开放，但需要支付/订阅/应用内购买
- [ ] 应用/干预功能已无法访问
- [ ] 其他:

主要医学适应症/疾病/病况 *

例如，“压力”、“糖尿病”，或在疾病后括号内定义目标群体，例如“自闭症（儿童的父母)”、“阿尔茨海默病患者（非正式照顾者)”

“laparoscopy or combined hysteroscopy for be

试验中测量的主要结局 *

试验中报告的主要结局列表以逗号分隔

“The change in pain scores between 6 and 7 h

次要/其他结局

干预措施还可能影响其他结果吗?

“Maximum pain score, anxiety score changes, length of hospital stay, hospitalization costs, and occurrence of adverse events”

?

推荐“剂量” *

用户使用说明中关于应用使用频率的说明是怎么说的?

- ○ 大约每天
- ○ 大约每周一次
- ○ 大约每月
- ○ 大约每年
- ◉ “按需”
- ○ 其他:

大约3个月后，按推荐使用该应用的用户（初学者）比例 *

- ◉ 未知/未评估
- ○ 0-10%
- ○ 11-20%
- ○ 21-30%
- ○ 31-40%
- ○ 41-50%
- ○ 51-60%
- ○ 61-70%
- ○ 71%-80%
- ○ 81-90%
- ○ 91-100%
- ○ 其他:

总体来说，这个应用/干预有效吗？ *

- ○ 是的：所有主要结局在干预组中均显著优于对照组
- ○ 部分：干预组与对照组的主要结局显著优异
- ◉ 对照组与干预组无统计学显著差异
- ○ 潜在有害：在一项或多项结局中，控制显著优于干预组
- ○ 结论不明确：需要更多研究
- ○ 其他:

论文准备状态/阶段 *

你目前处于文章准备的哪个阶段（填写此表格时）

- ○ 尚未提交——处于早期草稿状态
- ○ 尚未提交——处于晚稿状态，提交前不久
- ○ 已投稿但尚未审稿
- ◉ 提交给期刊并收到初步评审意见后
- ○ 已投稿并被接受，但尚未发表
- ○ 已发布
- ○ 其他:

期刊 *

如果您已经知道将提交本文（或已提交），请提供期刊名称（如果不是JMIR，请在“其他”栏填写期刊名称）

- ○ 尚未提交/不确定该在哪里提交
- ○ 医学互联网研究杂志（JMIR）
- ○ JMIR 移动健康与 UHealth
- ○ JMIR 严肃游戏
- ○ JMIR心理健康
- ○ JMIR公共卫生
- ◉ JMIR形成性研究
- ○ 其他JMIR姊妹期刊
- ○ 其他:

这是全功率效能试验还是试点/可行性试验？ *

- ◉ 试点/可行性
- ○ 全功率

手稿追踪号码 *

如果这是JMIR投稿，请在“其他”栏目中提供手稿追踪号码（ms追踪号码可在投稿确认邮件中，或在JMIR登录作者时找到）。如果论文已发表在JMIR，则ms追踪编号即为DOI末尾的四位数字，位于JMIR每篇发表文章底部）

- ○ 尚无MS编号（尚未）/未提交/发表于JMIR
- ◉ 其他: 92442

标题与摘要

?

1a）标题：标题中标注为随机试验

1a）你的论文是否涉及CONSORT第1a项？ *
也就是说，标题里有没有“随机对照试验”这个词？（如果没有，请在“其他”栏目下解释原因）

- [x] 是的
- [ ] 其他:

1a-i）标题中标明交付方式
确定交付方式。最好在标题中使用“基于网页”和/或“移动端”和/或“电子游戏”。避免使用“在线”、“虚拟”、“互动”等模糊词汇。仅当干预包含非基于网络的互联网组件（如电子邮件）时，使用“基于互联网”的称谓;仅在使用离线产品时使用“基于计算机”或“电子”。仅在“虚拟现实”(3D世界）的语境中使用“虚拟”。“在线”一词只在“在线支持小组”的语境中使用。用更广泛的产品类别术语来补充或替代产品名称（例如“移动”或“智能手机”代替“iphone”），尤其是在应用运行于不同平台时。

	1	2	3	4	5	
子项一点也不重要	○	○	○	○	●	必不可少

清除所选内容

你的纸质上有提到子项1a-i吗？ *
复制粘贴手稿标题中的相关部分（在引号中加上“类似”的引号以表示直接引用），或通过提供手稿中未包含的信息来详细说明该项目，或简要说明该项目为何不适用/不相关

“Effects of Virtual Reality on Postoperative Pain Following Minimally Invasive Gynecologic Surgery: A Randomized Controlled Trial”

1a-ii） 非基于网络的组成部分或标题中的重要共介入

如有，标题中提及非网络组件或重要的共同干预（例如“电话支持”）。

	1	2	3	4	5	
子项一点也不重要	○	○	○	○	○	必不可少

你的纸质上有提到子项1a-ii吗?

复制粘贴手稿标题中的相关部分（在引号中加上“类似”的引号以表示直接引用），或通过提供手稿中未包含的信息来详细说明该项目，或简要说明该项目为何不适用/不相关

您的回答

1a-iii）标题中的主要病症或目标群体

如有，请在标题中注明主要疾病或目标群体（例如，“针对1型糖尿病儿童”）。示例：一项基于网络和移动的电话干预，针对1型糖尿病儿童：随机对照试验

	1	2	3	4	5	
子项一点也不重要	○	○	○	○	◉	必不可少

清除所选内容

你的论文是否涉及1a-iii子项？ *

复制粘贴手稿标题中的相关部分（在引号中加上“类似”的引号以表示直接引用），或通过提供手稿中未包含的信息来详细说明该项目，或简要说明该项目为何不适用/不相关

“Effects of Virtual Reality on Postoperative Pain Following Minimally Invasive Gynecologic Surgery: A Randomized Controlled Trial”

1b） **摘要：试验设计、方法、结果和结论的结构化摘要**

NPT扩展：描述实验治疗、对照对象、护理提供者、中心及盲法状态。

1b-i） 摘要中方法部分中干预和比较器的关键特征/功能/组成部分

在摘要中提及干预和比较器的关键特征/功能/组成部分。如果可能，也提及设计网站时使用的理论和原则。考虑系统综述者和索引者的需求，包含重要的同义词。（注：仅在摘要中报道主报报道的内容。如果正文中缺少这些信息，请考虑添加）

	1	2	3	4	5	
子项一点也不重要	○	○	○	○	◉	必不可少

清除所选内容

你的纸质上有提到子项1b-i吗？ *

复制粘贴手稿摘要中的相关部分（在引号中加上“类似”的引号，以表示直接引用），或通过提供手稿中未包含的信息来详细说明，或简要说明该项目为何不适用/不相关

“The control group received conventional analgesic therapy after surgery, and the VR group received a 20-minute VR intervention at 6 hours postoperatively. ”

1b-ii） 摘要中方法部分的人类参与程度

澄清摘要中的人类参与程度，例如使用“全自动化”与“治疗师/护士/护理提供者/医生协助”等说法（如有涉及提供者的数量和专业能力）。（注：仅在摘要中报道主报报道的内容。如果正文中缺少这些信息，请考虑添加）

	1	2	3	4	5	
子项一点也不重要	○	○	○	○	○	必不可少

你的纸质上是否标注了1b-ii子项？

复制粘贴手稿摘要中的相关部分（在引号中加上“类似”的引号，以表示直接引用），或通过提供手稿中未包含的信息来详细说明，或简要说明该项目为何不适用/不相关

您的回答

1b-iii） 开放式与封闭式、基于网络（自我评估）与面对面评估，见摘要方法部分

请说明参与者的招募方式（线上与线下），例如来自开放获取网站、诊所或封闭在线用户组（封闭用户组试验），并澄清该试验是否纯为网络试验，或包含面对面环节（作为干预的一部分或评估）。明确说明结局是否通过问卷自我评估（如网络试验常见）。注意：在传统的线下试验中，开放试验（开放标签试验）是一种临床试验，研究人员和参与者都知道所用治疗方案。为避免混淆，应使用“盲法”或“非盲法”来表示盲法程度，而非“开放”，因为网络试验中的“开放”通常指“开放获取”（即参与者可以自行报名）。（注：仅在摘要中报道主报报道的内容。如果正文中缺少这些信息，请考虑添加）

	1	2	3	4	5	
子项一点也不重要	○	○	○	○	○	必不可少

你的论文是否涉及1b-iii子项？

复制粘贴手稿摘要中的相关部分（在引号中加上“类似”的引号，以表示直接引用），或通过提供手稿中未包含的信息来详细说明，或简要说明该项目为何不适用/不相关

您的回答

1b-iv） 摘要中的结果部分必须包含使用数据

报告各组参与者数量、干预措施的使用/接受情况（例如，流失/依从性指标、使用时间、登录次数等），以及主要/次要结局。（注：仅在摘要中报道主报报道的内容。如果正文中缺少这些信息，请考虑添加）

	1	2	3	4	5	
子项一点也不重要	○	○	○	○	●	必不可少

清除所选内容

你的论文是否涉及1b-iv子项?

复制粘贴手稿摘要中的相关部分（在引号中加上“类似”的引号，以表示直接引用），或通过提供手稿中未包含的信息来详细说明，或简要说明该项目为何不适用/不相关

“A total of 131 patients undergoing laparoscopy or combined hysteroscopy for benign gynecological diseases were randomly assigned (1:1) to either a VR group (n=68) or a control group (n=63). ” “The primary outcome was the change in pain scores between 6 and 7 hours. Secondary outcomes included maximum pain score, anxiety score changes, length of hospital stay, hospitalization costs, and occurrence of adverse events. Analyses were performed according to the intention-to-treat principle.”In this study, all participants received interventions according to the study protocol.

1b-v） 阴性试验的摘要结论/讨论

阴性试验的摘要结论/讨论：讨论主要结局——如果试验为阴性（主要结局未改变），且未使用干预措施，讨论阴性结果是否归因于缺乏采纳率及原因。（注：仅在摘要中报道主报报道的内容。如果正文中缺少这些信息，请考虑添加）

	1	2	3	4	5	
子项一点也不重要	○	○	○	○	○	必不可少

你的纸质文件里有提到1b-v子项吗?

复制粘贴手稿摘要中的相关部分（在引号中加上“类似”的引号，以表示直接引用），或通过提供手稿中未包含的信息来详细说明，或简要说明该项目为何不适用/不相关

您的回答

引言

2a）引言：科学背景及理论解释

?

2a-i） 问题及系统/解决方案类型

请描述问题及研究对象的系统/解决方案类型：是作为独立干预还是纳入更广泛的医疗项目？是针对特定患者群体的吗？干预的目标，例如，比其他干预更具成本效益，是替代还是补充其他解决方案？（注：干预措施的详细信息见第5条“方法”栏目）

	1	2	3	4	5	
子项一点也不重要	○	○	○	○	◉	必不可少

清除所选内容

你的纸质上有提到2a-i子项吗？ *

复制粘贴手稿中相关部分（在引号中加上“类似”的引号，以表示直接引用），或者通过提供手稿中未包含的信息来详细说明该项目，或简要说明该项目为何不适用/不相关

“The primary research purpose was to evaluate whether a single 20-minute VR intervention at 6 hours postoperatively can further reduce pain and anxiety compared with standard analgesia in patients undergoing minimally invasive gynecological surgery. The secondary purposes were to assess the effects of VR on hospital stay, hospitalization cost and adverse events, and to assess the safety and feasibility of VR in clinical practice. ”

2a-ii） 科学背景与理由：已知系统类型

科学背景与理由：关于研究对象（类型）系统的已知情况（如适用，务必讨论类似系统在其他疾病/诊断中的应用情况）、研究动机，即本研究的原因及背景，研究从利益相关者的角度出发， 发现的潜在影响[2]。简要说明选择比较对象的合理性。

	1	2	3	4	5	
子项一点也不重要	○	○	○	○	◉	必不可少

清除所选内容

你的论文是否涉及2a-ii子项？ *

复制粘贴手稿中相关部分（在引号中加上“类似”的引号，以表示直接引用），或者通过提供手稿中未包含的信息来详细说明该项目，或简要说明该项目为何不适用/不相关

“Virtual reality (VR) provides a three-dimensional immersive experience and has been reported to reduce pain and anxiety by diverting attention in various acute pain scenarios [5,6]. A recent meta-analysis including 92 randomized controlled trials and 7133 participants showed that immersive VR can effectively reduce pain perception during invasive medical operations [7]. Potential benefits of VR have also been reported in chronic pain management, physical rehabilitation, mental health treatment, oral surgery, and pediatric burn care [8-12]. However, high-quality evidence of VR for postoperative pain and anxiety after minimally invasive gynecological surgery is still insufficient [13]. Previous studies evaluating VR interventions in perioperative settings have been limited by small sample sizes, heterogeneous intervention protocols, and inconsistent outcome measures [14]. The optimal timing, duration and frequency of VR application in this population remain unclear. ”

2b）引言：具体目标或假设

你的论文是否涉及CONSORT子项2b？ *

复制粘贴手稿中相关部分（在引号中加上“类似”的引号，以表示直接引用），或者通过提供手稿中未包含的信息来详细说明该项目，或简要说明该项目为何不适用/不相关

“We hypothesized that VR might provide additional non pharmacological analgesic and anxiolytic benefits without increasing medical burden.”

方法

3a）试验设计的描述（如并行设计、阶乘设计），包括分配比率

你的论文是否涉及CONSORT子项3a? *

复制粘贴手稿中相关部分（在引号中加上“类似”的引号，以表示直接引用），或者通过提供手稿中未包含的信息来详细说明该项目，或简要说明该项目为何不适用/不相关

“The study was an exploratory study design. ””Participants were randomly assigned to either the VR or control group using a computer-generated random sequence prepared by an independent statistician. ”The allocation ratio is 1:1.

3b）试验开始后方法发生的重要变更（如资格标准），并附有原因

你的论文是否涉及CONSORT子项3b? *

复制粘贴手稿中相关部分（在引号中加上“类似”的引号，以表示直接引用），或者通过提供手稿中未包含的信息来详细说明该项目，或简要说明该项目为何不适用/不相关

No significant changes were made to the method after the trial commenced.

3b-i） 修复漏洞、停机、内容变更

修复漏洞、停机、内容变更：电子健康系统通常是动态的。因此，方法变更的描述还包括试验期间对干预或比较器所做的重要变更（例如重大漏洞修复或功能或内容的变更）（5-iii）以及其他可能影响研究设计的“意外事件”，如人员变动、系统故障/停机等[2]。

	1	2	3	4	5	
子项一点也不重要	○	○	○	○	○	必不可少

你的纸质上有提到3b-i子项吗?

复制粘贴手稿中相关部分（在引号中加上“类似”的引号，以表示直接引用），或者通过提供手稿中未包含的信息来详细说明该项目，或简要说明该项目为何不适用/不相关

您的回答

4a）参与者资格标准

你的论文是否涉及CONSORT子项4a? *

复制粘贴手稿中相关部分（在引号中加上"类似"的引号，以表示直接引用），或者通过提供手稿中未包含的信息来详细说明该项目，或简要说明该项目为何不适用/不相关

"Inclusion criteria:
(1) Nonpregnant women aged 18–70 years, admitted for benign gynecological diseases and scheduled to undergo laparoscopy or combined hysteroscopy.
(2) Willingness to participate in the study.
(3) Ability to read and independently complete questionnaires.
Exclusion criteria:
Patients were excluded if they had suspected malignancy, anatomical abnormalities, severe comorbidities, sensory impairments, unwillingness to participate, or a history of more than three prior abdominal surgeries.
Exit criteria:
(1) Serious complications occurred during the study.
(2) The operation was cancelled for any reason.
(3) Severe intolerance to pain.
(4) The patient requested to withdraw from the study."

4a-i） 计算机/互联网素养

计算机/互联网素养通常是隐含的"事实上"资格标准——这一点应当明确说明。

	1	2	3	4	5	
子项一点也不重要	○	○	○	○	○	必不可少

你的纸质上有提到4a-i子项目吗?

复制粘贴手稿中相关部分（在引号中加上"类似"的引号，以表示直接引用），或者通过提供手稿中未包含的信息来详细说明该项目，或简要说明该项目为何不适用/不相关

您的回答

4a-ii） 开放式与封闭式、基于网络与面对面的评估：

开放式与闭式、基于网络与面对面的评估：说明参与者的招募方式（在线与线下），例如通过开放获取网站或诊所，并澄清此次试验是否纯粹基于网络，还是包含面对面环节（作为干预的一部分或评估），即研究团队对参与者的了解程度如何。在仅在线试验中，应明确参与者是否为准匿名，是否可能拥有多重身份，或是否使用技术或后勤措施（如Cookie、电子邮件确认、电话）来检测或防止此类情况。

	1	2	3	4	5	
子项一点也不重要	○	○	○	○	◉	必不可少

清除所选内容

你的纸质文件里有提到4a-ii子项吗？ *

复制粘贴手稿中相关部分（在引号中加上"类似"的引号，以表示直接引用），或者通过提供手稿中未包含的信息来详细说明该项目，或简要说明该项目为何不适用/不相关

"Eligible patients were consecutively screened and recruited from the Department of Obstetrics and Gynecology at Sun Yat-sen Memorial Hospital of Sun Yat-sen University between April 2021 and April 2022. "

4a-iii） 招募期间的信息传递

招聘时提供的信息。说明参与者在招募和知情同意程序中如何被简报（例如，将知情同意文件作为附录发布，另见X26项），因为这些信息可能影响用户的自我选择、用户期望，并可能影响结果偏差。

	1	2	3	4	5	
子项一点也不重要	○	○	○	○	○	必不可少

你的论文是否涉及4a-iii子项？

复制粘贴手稿中相关部分（在引号中加上"类似"的引号，以表示直接引用），或者通过提供手稿中未包含的信息来详细说明该项目，或简要说明该项目为何不适用/不相关

您的回答

4b）数据采集的环境和地点

你的论文是否涉及CONSORT子项4b？ *

复制粘贴手稿中相关部分（在引号中加上“类似”的引号，以表示直接引用），或者通过提供手稿中未包含的信息来详细说明该项目，或简要说明该项目为何不适用/不相关

" Patients in the VR group received a 20-minute VR session in the gynecology ward 6 hours post-surgery, utilizing portable standalone VR glasses with 3D dynamic content. "

4b-i）报告结局是否通过在线问卷进行（自我）评估

明确报告结局是否通过在线问卷（如网络试验常见）进行（自我）评估。

	1	2	3	4	5	
子项一点也不重要	○	○	○	○	◉	必不可少

清除所选内容

你的纸质上有写子项4b-i吗？ *

复制粘贴手稿中相关部分（在引号中加上“类似”的引号，以表示直接引用），或者通过提供手稿中未包含的信息来详细说明该项目，或简要说明该项目为何不适用/不相关

The research results were obtained through self-assessment using paper questionnaires.

4b-ii） 报告机构隶属关系的显示方式

报告机构隶属关系如何向潜在参与者显示[电子健康媒体]，因为与知名医院或大学的关联可能影响志愿者参与率、使用率及干预反应。（非必修项目——仅在可能影响结果时描述）

	1	2	3	4	5	
子项一点也不重要	○	○	○	○	○	必不可少

你的纸质上是否涉及4b-ii子项？

复制粘贴手稿中相关部分（在引号中加上“类似”的引号，以表示直接引用），或者通过提供手稿中未包含的信息来详细说明该项目，或简要说明该项目为何不适用/不相关

您的回答

5）每组干预措施需具备足够细节以便重复，包括实际实施方式和时间

5-i） 提及开发商、赞助商和所有者的姓名、资质、隶属关系

提及开发者、赞助商和所有者的姓名、资质、隶属关系[6]（如果作者/评估者是软件的所有者或开发者，则需在“利益冲突”部分声明或在稿件其他地方注明）。

	1	2	3	4	5	
子项一点也不重要	○	○	○	○	○	必不可少

你的论文是否涉及5-i子项？

复制粘贴手稿中相关部分（在引号中加上“类似”的引号，以表示直接引用），或者通过提供手稿中未包含的信息来详细说明该项目，或简要说明该项目为何不适用/不相关

您的回答

5-ii）描述历史/开发过程

描述应用的历史/开发过程以及以往的形成性评估（如焦点小组、可用性测试），因为这些将影响采用率/使用率，并有助于解读结果。

	1	2	3	4	5	
子项一点也不重要	○	○	○	○	○	必不可少

你的纸质上有提到子项5-ii吗？

复制粘贴手稿中相关部分（在引号中加上“类似”的引号，以表示直接引用），或者通过提供手稿中未包含的信息来详细说明该项目，或简要说明该项目为何不适用/不相关

您的回答

5-iii）修订与更新

修订和更新。明确说明评估的应用/干预（及对照组，如适用）的日期和/或版本号，或描述干预在评估过程中是否发生重大变化，或试验期间开发和/或内容是否“冻结”。描述动态组件，如新闻推送或内容变化，这些可能影响干预的可重复性（关于意外事件，见第3b项）。

	1	2	3	4	5	
子项一点也不重要	○	○	○	○	○	必不可少

你的论文是否涉及第5-iii项子项？

复制粘贴手稿中相关部分（在引号中加上“类似”的引号，以表示直接引用），或者通过提供手稿中未包含的信息来详细说明该项目，或简要说明该项目为何不适用/不相关

您的回答

5-iv）质量保证方法

如适用，提供质量保证方法的信息，以确保信息的准确性和质量[1]。

	1	2	3	4	5	
子项一点也不重要	○	○	○	○	○	必不可少

你的论文是否涉及第5-iv项子项？

复制粘贴手稿中相关部分（在引号中加上“类似”的引号，以表示直接引用），或者通过提供手稿中未包含的信息来详细说明该项目，或简要说明该项目为何不适用/不相关

您的回答

5-v）通过发布源代码和/或提供截图/截图视频和/或算法流程图来确保可复制性

通过发布源代码和/或提供截图/截图视频和/或算法流程图来确保可复制性。可重复性（即原则上其他研究者应能够复制该研究）是科学报道的标志。

	1	2	3	4	5	
子项一点也不重要	○	○	○	○	○	必不可少

你的纸质上有提到子项5-v吗？

复制粘贴手稿中相关部分（在引号中加上“类似”的引号，以表示直接引用），或者通过提供手稿中未包含的信息来详细说明该项目，或简要说明该项目为何不适用/不相关

您的回答

5-vi）数字保存

数字保存：提供申请的网址，但由于干预措施可能会随着时间变化或消失;同时确保干预内容被存档（互联网档案馆、webcitation.org，和/或与文章一同发布源代码、截图/视频）。由于登录界面后面的页面无法归档，建议创建无需登录即可访问的演示页面。

	1	2	3	4	5	
子项一点也不重要	○	○	○	○	○	必不可少

你的纸上有提到子项5-vi吗？

复制粘贴手稿中相关部分（在引号中加上“类似”的引号，以表示直接引用），或者通过提供手稿中未包含的信息来详细说明该项目，或简要说明该项目为何不适用/不相关

您的回答

5-vii） 访问

访问性：描述参与者如何访问申请，在何种环境/情境下，是否必须付费（或被付费），是否必须是特定群体成员。如果已知，请描述参与者如何获得“平台和互联网访问”[1]。为确保编辑/审稿人/读者访问，建议提供“后门”登录账户或演示模式，供审稿人/读者探索应用（对存档目的也很重要，见vi）。

	1	2	3	4	5	
子项一点也不重要	○	○	○	○	◉	必不可少

清除所选内容

你的论文是否涉及第5-vii子项？ *

复制粘贴手稿中相关部分（在引号中加上“类似”的引号，以表示直接引用），或者通过提供手稿中未包含的信息来详细说明该项目，或简要说明该项目为何不适用/不相关

"All patients scheduled for laparoscopic or combined hysteroscopic surgery for benign gynecological conditions were assessed for eligibility according to predefined inclusion and exclusion criteria. "Patients did not need to pay.

5-viii） 传递方式、干预和比较器的特征/功能/组成部分，以及理论框架

描述实施方式、干预和比较器的特征/功能/组成部分，以及用于设计它们的理论框架[6]（教学策略[1]、行为改变技巧、说服特征等，术语见例如[7， 8]）。内容包括对内容的深入描述（包括内容来源及开发者）[1]“，是否[以及如何]针对个别情况量身定制，并允许用户跟踪进度并获得反馈”[6]。这还包括通信传递渠道的描述，以及——如果计算机介导通信是其中一部分——通信是同步还是异步[6]。它还包括关于展示策略的信息[1]，包括页面设计原则、页面平均文本量、指向其他资源的超链接存在等[1]。

	1	2	3	4	5	
子项一点也不重要	○	○	○	○	◉	必不可少

清除所选内容

?

你的论文是否涉及第5-viii项子项？ *

复制粘贴手稿中相关部分（在引号中加上“类似”的引号，以表示直接引用），或者通过提供手稿中未包含的信息来详细说明该项目，或简要说明该项目为何不适用/不相关

"The control group received conventional analgesic drugs for pain relief, and the VR group received only VR technology without any analgesic drugs. Patients in the VR group received a 20-minute VR session in the gynecology ward 6 hours post-surgery, utilizing portable standalone VR glasses with 3D dynamic content. "

5-ix）描述使用参数

描述使用参数（例如预期“剂量”和最佳使用时机）。明确向用户提供了哪些指示或建议，例如关于使用时间、频率、使用频率（如有）或干预是否是随意使用的。

	1	2	3	4	5	
子项一点也不重要	○	○	○	○	○	必不可少

你的论文是否涉及第5-ix项子项?

复制粘贴手稿中相关部分（在引号中加上“类似”的引号，以表示直接引用），或者通过提供手稿中未包含的信息来详细说明该项目，或简要说明该项目为何不适用/不相关

您的回答

5-x） 明确人类参与的程度

明确电子干预或协同干预中的人类参与程度（包括护理人员或医疗专业人员，以及技术援助）（如有相关专业人员的详细信息和专业能力，以及“提供的援助类型、支持的时间和频率、启动方式以及援助的提供方式”。可能需要区分试验所需的人类参与程度与RCT环境外常规应用所需的人类参与程度（详见第21项——可推广性）。

	1	2	3	4	5	
子项一点也不重要	○	○	○	○	○	必不可少

你的纸质上是否涉及子项5-x?

复制粘贴手稿中相关部分（在引号中加上“类似”的引号，以表示直接引用），或者通过提供手稿中未包含的信息来详细说明该项目，或简要说明该项目为何不适用/不相关

您的回答

5-习） 报告任何提示/提醒

报告任何使用提示/提醒：澄清是否有提示（信件、邮件、电话、短信）提示使用应用，触发因素、频率等。可能需要区分试验所需的提示/提醒程度，以及RCT环境外常规应用的提示/提醒程度（详见第21项——可推广性）。

	1	2	3	4	5	
子项一点也不重要	◉	○	○	○	○	必不可少

清除所选内容

你的论文是否涉及第5-习项? *

复制粘贴手稿中相关部分（在引号中加上“类似”的引号，以表示直接引用），或者通过提供手稿中未包含的信息来详细说明该项目，或简要说明该项目为何不适用/不相关

No prompts or reminders are required in this study.

5-xii） 描述任何共同干预（包括培训/支持）

描述任何共同干预（包括培训/支持）：明确说明除针对性电子健康干预外提供的任何干预措施，因为电子健康干预可能不设计为独立干预。这包括培训课程和支持[1]。可能需要区分试验所需的培训水平与RCT环境外常规应用的培训水平（详见第21项——可推广性）。

	1	2	3	4	5	
子项一点也不重要	○	○	○	○	◉	必不可少

清除所选内容

你的论文是否涉及子项5-xii? *

复制粘贴手稿中相关部分（在引号中加上“类似”的引号，以表示直接引用），或者通过提供手稿中未包含的信息来详细说明该项目，或简要说明该项目为何不适用/不相关

" All patients received a standardized general anesthesia protocol intraoperatively. "Patients in the virtual reality group received a 20-minute virtual reality intervention 6 hours after surgery, with no combined interventions applied." If the patient has severe pain, the corresponding analgesic regimen will be given immediately according to clinical need. Such patients were withdrawn from the intervention protocol to reduce potential confounding."

6a）完全定义的预先指定的主要和次要结局指标，包括评估方式和时间

你的论文是否涉及CONSORT子项6a? *

复制粘贴手稿中相关部分（在引号中加上“类似”的引号，以表示直接引用），或者通过提供手稿中未包含的信息来详细说明该项目，或简要说明该项目为何不适用/不相关

"The primary outcome was the improvement in Visual Analog Scale (VAS) score for pain at 7 and 6 hours postoperatively. The VAS scores are divided into 0 to 10 points according to the number scale. 0 indicates no pain, anxiety, etc., and 10 indicates unbearable severe pain, anxiety, etc. Secondary outcomes included the maximum pain score, improvement in anxiety levels assessed using the VAS for anxiety. Total length of stay, total hospitalization cost and adverse events such as dizziness, nausea, and vomiting were also recorded in each group. "

6a-i） 在线问卷：说明其是否经过在线使用验证，并应用CHERRIES题目来描述问卷的设计和部署情况

如果结果是通过在线问卷获得的，请说明是否经过在线使用验证，并应用CHERRIES题目来描述问卷的设计和部署方式[9]。

	1	2	3	4	5	
子项一点也不重要	○	○	○	○	○	必不可少

你的纸质文件里有提到6a-i子项吗?

从手稿文本中复制粘贴相关章节

您的回答

6a-ii） 描述“使用”(包括使用强度/剂量）是否以及如何被定义/测量/监测

描述“使用”(包括使用强度/剂量）是否以及如何被定义/测量/监控（登录、日志分析等)。使用/采用指标是重要的流程结果，任何电子健康试验都应当报告。

	1	2	3	4	5	
子项一点也不重要	○	○	○	○	○	必不可少

你的纸质上有提到6a-ii子项吗?

从手稿文本中复制粘贴相关章节

您的回答

6a-iii）描述是否、如何以及何时从参与者那里获得了定性反馈

描述是否、如何以及何时从参与者那里获得定性反馈（例如通过电子邮件、反馈表、访谈、焦点小组)。

	1	2	3	4	5	
子项一点也不重要	○	○	○	○	○	必不可少

你的论文是否涉及6a-iii子项?

从手稿文本中复制粘贴相关章节

您的回答

6b）试验开始后对结果的任何变化及其原因

你的论文是否涉及CONSORT子项6b？ *

复制粘贴手稿中相关部分（在引号中加上“类似”的引号，以表示直接引用），或者通过提供手稿中未包含的信息来详细说明该项目，或简要说明该项目为何不适用/不相关

No changes occurred to trial outcomes after the study initiation.

7a）样本量的确定方式

NPT：在适用的情况下，详细说明是否以及如何处理照护机构或中心的分组问题

7a-i）描述在计算样本量时是否以及如何考虑预期的淘汰率

描述在计算样本量时是否以及如何考虑预期的流失情况。

	1	2	3	4	5	
子项一点也不重要	○	○	○	○	◉	必不可少

清除所选内容

你的纸质上有提到7a-i子项吗？

复制粘贴手稿标题中的相关部分（在引号中加上“类似”的引号以表示直接引用），或通过提供手稿中未包含的信息来详细说明该项目，或简要说明该项目为何不适用/不相关

"To account for an anticipated 20% dropout rate, we planned to recruit at least 39 participants per group, for a total of 78 patients. "

7b） 在适用的情况下，解释任何中期分析和停止指导原则

你的论文是否涉及CONSORT子项7b？ *

复制粘贴手稿中相关部分（在引号中加上“类似”的引号，以表示直接引用），或者通过提供手稿中未包含的信息来详细说明该项目，或简要说明该项目为何不适用/不相关

"To promote patient safety and ethical compliance, the researchers closely monitored the patients in the virtual reality group. If the patient has severe pain, the corresponding analgesic regimen will be given immediately according to clinical need. Such patients were withdrawn from the intervention protocol to reduce potential confounding."

8a）生成随机分配序列的方法

NPT：在适用的情况下，护理提供者如何分配给每个试验组

你的论文是否涉及CONSORT子项8a？ *

复制粘贴手稿中相关部分（在引号中加上“类似”的引号，以表示直接引用），或者通过提供手稿中未包含的信息来详细说明该项目，或简要说明该项目为何不适用/不相关

"Participants were randomly assigned to either the VR or control group using a computer-generated random sequence prepared by an independent statistician. Sequentially numbered, sealed, opaque envelopes were used to implement distribution and concealment. Only after the research coordinator obtained the informed consent of the patient can the corresponding envelope be opened."

8b）随机化类型;任何限制的细节（如区块和区块大小）

你的论文是否涉及CONSORT子项8b？ *

复制粘贴手稿中相关部分（在引号中加上“类似”的引号，以表示直接引用），或者通过提供手稿中未包含的信息来详细说明该项目，或简要说明该项目为何不适用/不相关

Patients undergoing laparoscopy or combined hysteroscopy for benign gynecological diseases were randomly divided into the virtual reality group and the control group at a 1:1 ratio.

9）用于实现随机分配序列（如顺序编号容器）的机制，描述在分配干预前所采取的隐藏序列步骤

你的论文是否涉及CONSORT子项9？ *

复制粘贴手稿中相关部分（在引号中加上"类似"的引号，以表示直接引用），或者通过提供手稿中未包含的信息来详细说明该项目，或简要说明该项目为何不适用/不相关

"Participants were randomly assigned to either the VR or control group using a computer-generated random sequence prepared by an independent statistician. Sequentially numbered, sealed, opaque envelopes were used to implement distribution and concealment. Only after the research coordinator obtained the informed consent of the patient can the corresponding envelope be opened. "

10）谁制定了随机分配序列，谁招募了参与者，谁将参与者分配到干预项目

你的论文是否涉及CONSORT子项10？ *

复制粘贴手稿中相关部分（在引号中加上"类似"的引号，以表示直接引用），或者通过提供手稿中未包含的信息来详细说明该项目，或简要说明该项目为何不适用/不相关

Core researchers of this study were responsible for generating random allocation sequences, recruiting participants and assigning them to intervention groups.

11a）如果进行，谁在分配干预后被盲法（例如参与者、护理提供者、评估结果者）以及如何进行

NPT：是否盲视了共同干预的组别分配

11a-i）明确谁被失明，谁没有

具体说明谁被失明，谁没有。通常，在基于网络的试验中，参与者无法盲法[1， 3]（这一点应明确说明），但可能对结果评估者、数据分析人员或进行联合干预（如有）进行盲法。

	1	2	3	4	5	
子项一点也不重要	○	○	○	○	◉	必不可少

清除所选内容

你的纸上有提到11a-i子项吗？ *

复制粘贴手稿中相关部分（在引号中加上“类似”的引号，以表示直接引用），或者通过提供手稿中未包含的信息来详细说明该项目，或简要说明该项目为何不适用/不相关

"Due to the nature of VR therapy, patients and healthcare providers were aware of the intervention plan, but outcome assessors who collected postoperative pain and anxiety data were blinded to group allocation. This approach reduced the risk of assessment bias and improved methodological rigor of the trial. "

11a-ii） 讨论例如，参与者是否知道哪种干预是“感兴趣的干预”，哪种是“对照”。

知情同意程序（4a-ii）可能产生偏见和某些预期——例如，参与者是否知道哪种干预是“感兴趣的干预”，哪种是“对照”。

	1	2	3	4	5	
子项一点也不重要	○	○	○	○	○	必不可少

你的纸质上有提到11a-ii子项目吗?

复制粘贴手稿中相关部分（在引号中加上“类似”的引号，以表示直接引用），或者通过提供手稿中未包含的信息来详细说明该项目，或简要说明该项目为何不适用/不相关

您的回答

11b）如有需要，描述干预措施的相似性

（该项通常与电子健康试验无关，因为它涉及安慰剂或假干预与有效药物/干预措施的相似性）

你的论文是否涉及CONSORT子项11b？ *

复制粘贴手稿中相关部分（在引号中加上“类似”的引号，以表示直接引用），或者通过提供手稿中未包含的信息来详细说明该项目，或简要说明该项目为何不适用/不相关

Due to the characteristics of virtual reality therapy, both patients and medical staff were aware of the intervention protocols in the VR group and control group. However, the assessors collecting postoperative pain and anxiety data were blinded to group allocation.

12a）用于比较主要与次要结局组别的统计方法
NPT：在适用的情况下，详细说明护理提供者或中心是否以及如何处理了该群体问题

你的论文是否涉及CONSORT子项12a？ *
复制粘贴手稿中相关部分（在引号中加上“类似”的引号，以表示直接引用），或者通过提供手稿中未包含的信息来详细说明该项目，或简要说明该项目为何不适用/不相关

"The primary outcome was analyzed using a generalized linear model (GLM) with identity link function, including treatment group (VR vs control) as a fixed effect and baseline VAS score as a covariate. Adjusted mean differences with 95% confidence intervals (CIs) were estimated from the model.
Secondary continuous outcomes were analyzed using similar GLM approaches. For outcomes with a single post-baseline measurement, treatment group was included as the sole predictor. "

12a-i）处理遗失/缺失值的补算技术
处理流失/缺失值的补值技术：并非所有参与者都会按预期使用干预/比较工具，且电子健康试验中流失率通常较高。具体说明未使用应用或退出试验的参与者在统计分析中如何被对待（强烈建议进行完整病例分析，简单的补缺技术如LOCF也可能存在问题[4]）。

	1	2	3	4	5	
子项一点也不重要	○	○	○	○	○	必不可少

你的纸质文件里有提到子项12a-i吗？ *
复制粘贴手稿中相关部分（在引号中加上“类似”的引号，以表示直接引用），或者通过提供手稿中未包含的信息来详细说明该项目，或简要说明该项目为何不适用/不相关

No missing data were observed for primary or secondary outcomes.

12b）附加分析方法，如子组分析和调整分析

你的论文是否涉及CONSORT子项12b？ *

复制粘贴手稿中相关部分（在引号中加上"类似"的引号，以表示直接引用），或者通过提供手稿中未包含的信息来详细说明该项目，或简要说明该项目为何不适用/不相关

"The initial analysis was not corrected for confounders. Additionally, covariate-adjusted analyses were conducted for all outcomes, adjusting for age, type of surgery, number of previous surgeries, and gravidity. No formal within-group inferential analyses were prespecified. "

X26）REB/IRB批准与伦理考量[推荐作为"方法"小标题]（非CONSORT项目）

X26-i）对伦理委员会批准的评论

	1	2	3	4	5	
子项一点也不重要	○	○	○	○	○	必不可少

你的纸质上有写子项X26-i吗？

复制粘贴手稿中相关部分（在引号中加上"类似"的引号，以表示直接引用），或者通过提供手稿中未包含的信息来详细说明该项目，或简要说明该项目为何不适用/不相关

您的回答

x26-ii） 概述知情同意程序

概述知情同意程序，例如，如果同意是线下还是在线获得（如何获得？复选框等？），以及提供了哪些信息（参见4a-ii）。参见[6]，了解知情同意文件中将包含的一些内容。

	1	2	3	4	5	
子项一点也不重要	○	○	○	○	○	必不可少

你的纸上有写子项X26-ii吗?

复制粘贴手稿中相关部分（在引号中加上“类似”的引号，以表示直接引用），或者通过提供手稿中未包含的信息来详细说明该项目，或简要说明该项目为何不适用/不相关

您的回答

X26-iii） 安全与保安程序

安全与保障措施，包括隐私考虑，以及采取任何措施以降低伤害的可能性或被发现（例如，教育培训、热线的可用性）

	1	2	3	4	5	
子项一点也不重要	○	○	○	○	○	必不可少

你的纸质上有提到子项X26-iii吗?

复制粘贴手稿中相关部分（在引号中加上“类似”的引号，以表示直接引用），或者通过提供手稿中未包含的信息来详细说明该项目，或简要说明该项目为何不适用/不相关

您的回答

选举结果

13a） 每组随机分配、接受预期治疗并分析主要结局的参与者数量

NPT：每个组内执行干预的护理提供者或中心数量，以及每个护理提供者在该中心治疗的患者数量

你的论文是否涉及CONSORT子项13a? *

复制粘贴手稿中相关部分（在引号中加上“类似”的引号，以表示直接引用），或者通过提供手稿中未包含的信息来详细说明该项目，或简要说明该项目为何不适用/不相关

"A total of 143 patients were initially randomized. Twelve patients were excluded for not meeting inclusion criteria. All remaining patients completed the questionnaires during hospitalization, with no loss to follow-up. Therefore, 68 patients in the VR group and 63 in the control group were included in the final analysis (Figure 1)."

13b）每个组的随机分组后损失和排除情况及其原因

你的论文是否涉及CONSORT子项13b? （注：最好以CONSORT流程图显示） *

复制粘贴手稿中相关部分（在引号中加上“类似”的引号，以表示直接引用），或者通过提供手稿中未包含的信息来详细说明该项目，或简要说明该项目为何不适用/不相关

"A total of 143 patients were initially randomized. Twelve patients were excluded for not meeting inclusion criteria. "
"Age does not match(n=4)
Excessive number of previous surgeries(n=1)
Malignant tumor surgery(n=4)
Discrepancies in surgical methods(n=3)"

13b-i） 消耗图

强烈建议：使用流失图（例如，每组仍登录或使用干预/对照器的参与者比例，随时间绘制，类似生存曲线）或其他显示使用/剂量/参与的图表。

	1	2	3	4	5	
子项一点也不重要	○	○	○	○	○	必不可少

你的纸质上有提到子项13b-i吗?

复制粘贴手稿中相关部分，或如适用引用图号（如“这样”在引号内加引号以表示直接引用），或通过提供手稿中未包含的信息来详细说明该项目，或简要说明为何该项目不适用于/不相关

您的回答

14a）定义招募和跟进期间的日期

你的论文是否涉及CONSORT子项14a? *

复制粘贴手稿中相关部分（在引号中加上“类似”的引号，以表示直接引用），或者通过提供手稿中未包含的信息来详细说明该项目，或简要说明该项目为何不适用/不相关

"Eligible patients were consecutively screened and recruited from the Department of Obstetrics and Gynecology at Sun Yat-sen Memorial Hospital of Sun Yat-sen University between April 2021 and April 2022. "

14a-i）指出关键的“世俗事件”是否属于研究期内

指出是否涉及关键的“世俗事件”，例如互联网资源的重大变化或“计算机硬件或互联网传输资源的变化”

	1	2	3	4	5	
子项一点也不重要	○	○	○	○	○	必不可少

你的纸质上有提到子项14a-i吗?

复制粘贴手稿中相关部分（在引号中加上“类似”的引号，以表示直接引用），或者通过提供手稿中未包含的信息来详细说明该项目，或简要说明该项目为何不适用/不相关

您的回答

14b）审判为何提前结束或终止

你的论文是否涉及CONSORT子项14b？ *

复制粘贴手稿中相关部分（在引号中加上“类似”的引号，以表示直接引用），或者通过提供手稿中未包含的信息来详细说明该项目，或简要说明该项目为何不适用/不相关

The trial did not end or be terminated prematurely.

15）一组显示各组基线人口统计和临床特征的表格

NPT：如适用，描述各组的护理提供者（病例量、资质、专业水平等）及中心（数量）

你的论文是否涉及CONSORT子项15？ *

复制粘贴手稿中相关部分（在引号中加上“类似”的引号，以表示直接引用），或者通过提供手稿中未包含的信息来详细说明该项目，或简要说明该项目为何不适用/不相关

"Table 1. Baseline demographic and clinical characteristics of participants in VR group and control group. (N=131)"

15-i）报告与数字鸿沟问题相关的人口统计

在电子健康试验中，特别重要的是报告与数字鸿沟问题相关的人口统计数据，如年龄、教育程度、性别、社会经济状况、参与者的计算机/互联网/电子健康素养（如已知）。

	1	2	3	4	5	
子项一点也不重要	○	○	○	○	◉	必不可少

清除所选内容

你的纸质上有提到子项15-i吗？ *

复制粘贴手稿中相关部分（在引号中加上"类似"的引号，以表示直接引用)，或者通过提供手稿中未包含的信息来详细说明该项目，或简要说明该项目为何不适用/不相关

"Demographic and clinical baseline data of the subjects were collected at randomization, including age, pregnancy, menopausal status, number of previous operations and surgical methods. Before surgery, a baseline psychological state assessment was carried out. We used standardized scales that were validated for reliability and validity, including the Anxiety Self-Rating Scale (SAS), the Depression Self-Rating Scale (SDS), and the Pain Catastrophizing Scale (PCS). "

16）每个组的分析中包含的参与者人数（分母）以及分析是否按原始分配组划分

16-i） 报告多个"分母"并提供定义

报告多个"分母"并提供定义：报告N个（及其效应大小）"涵盖"在研究参与[和使用]阈值范围内"[1]，例如，N个暴露，N个同意，N个使用超过x次，N个使用超过y周，N个参与者在特定预定时间点（按绝对和相对人数)"使用"干预/对比器。始终明确定义干预的"用途"。

	1	2	3	4	5	
子项一点也不重要	○	○	○	○	◉	必不可少

清除所选内容

你的论文是否涉及子项16-i? *

复制粘贴手稿中相关部分（在引号中加上"类似"的引号，以表示直接引用)，或者通过提供手稿中未包含的信息来详细说明该项目，或简要说明该项目为何不适用/不相关

"A total of 143 patients were initially randomized. Twelve patients were excluded for not meeting inclusion criteria. All remaining patients completed the questionnaires during hospitalization, with no loss to follow-up. Therefore, 68 patients in the VR group and 63 in the control group were included in the final analysis. " The analysis was by original assigned groups.

16-ii）初级分析应为意图治疗

初级分析应为意图治疗，次级分析可仅比较“用户”，但需注意该样本不再是随机样本（见18-i）。

	1	2	3	4	5	
子项一点也不重要	○	○	○	○	○	必不可少

你的论文是否涉及第16-ii项？

复制粘贴手稿中相关部分（在引号中加上“类似”的引号，以表示直接引用），或者通过提供手稿中未包含的信息来详细说明该项目，或简要说明该项目为何不适用/不相关

您的回答

17a） **每个主要和次要结局、各组结果、估计效应大小及其精度（如95%置信区间）**

?

你的论文是否涉及CONSORT子项17a? *

复制粘贴手稿中相关部分（在引号中加上“类似”的引号，以表示直接引用），或者通过提供手稿中未包含的信息来详细说明该项目，或简要说明该项目为何不适用/不相关

"Primary Outcome
The primary outcome, defined as the improvement in VAS score between 6 and 7 hours postoperatively, did not differ significantly between the VR and control groups. In the unadjusted analysis, the mean difference was 0.169 (95% CI −0.271 to 0.608; P = 0.449). After adjustment for prespecified covariates (age, type of surgery, number of previous surgeries, and gravidity), the result remained non-significant (mean difference 0.136, 95% CI −0.300 to 0.573; P = 0.538) (Table 2).
Secondary Outcomes
No statistically significant differences were observed between the two groups for any secondary continuous outcomes. Maximum pain score showed a non-significant trend toward higher values in the VR group (unadjusted mean difference 0.839, 95% CI −0.101 to 1.779; P = 0.080; adjusted P = 0.098).
Improvement in anxiety scores (6–7 h) was comparable between groups (unadjusted mean difference 0.042, 95% CI −0.365 to 0.449; P = 0.838; adjusted P = 0.837).
Total length of hospital stay did not differ significantly (unadjusted mean difference −0.317 days, 95% CI −0.946 to 0.312; P = 0.321; adjusted P = 0.313).
Total hospitalization costs were also similar between groups (unadjusted mean difference 107.426 CNY, 95% CI −2314.644 to 2529.495; P = 0.930; adjusted P = 0.964) (Table 2).
Postoperative Symptoms
Postoperative symptom improvements, including dizziness, nausea, and vomiting, were analyzed as continuous variables. No statistically significant differences were found between groups:
Improvement in dizziness and nausea scores: mean difference −0.303 (95% CI −1.066 to 0.460; P = 0.433; adjusted P = 0.459)
Improvement in vomiting score: mean difference 0.147 (95% CI −0.108 to 0.401; P = 0.257; adjusted P = 0.265).
These findings suggest that VR intervention was not associated with significant improvements in postoperative symptom recovery compared to the control group (Table 2)."

17a-i） 流程结果的呈现，如使用指标和使用强度

除了主要/次要（临床）结局外，呈现过程结果如使用指标和使用强度（剂量、暴露）及其操作定义至关重要。这不仅指流失度量（13-b）（通常是二元变量），还包括更连续的暴露度量，如“平均会话时长”。这些必须附带技术描述，说明如何定义像“会话”这样的指标（例如，闲置时间后的超时）[1]（见第6a项报告）。

	1	2	3	4	5	
子项一点也不重要	○	○	○	○	○	必不可少

你的论文是否涉及17a-i子项?
复制粘贴手稿中相关部分（在引号中加上“类似”的引号，以表示直接引用），或者通过提供手稿中未包含的信息来详细说明该项目，或简要说明该项目为何不适用/不相关

您的回答

17b）对于二元结果，建议同时呈现绝对效应和相对效应量

你的论文是否涉及CONSORT子项17b? *
复制粘贴手稿中相关部分（在引号中加上“类似”的引号，以表示直接引用），或者通过提供手稿中未包含的信息来详细说明该项目，或简要说明该项目为何不适用/不相关

Absolute and relative effect sizes are presented in the analysis of this paper.

18）任何其他分析的结果，包括亚组分析和调整分析，区分预定分析与探索分析

你的论文是否涉及CONSORT子项18? *
复制粘贴手稿中相关部分（在引号中加上“类似”的引号，以表示直接引用），或者通过提供手稿中未包含的信息来详细说明该项目，或简要说明该项目为何不适用/不相关

"Postoperative Symptoms
Postoperative symptom improvements, including dizziness, nausea, and vomiting, were analyzed as continuous variables. No statistically significant differences were found between groups:
Improvement in dizziness and nausea scores: mean difference −0.303 (95% CI −1.066 to 0.460; P = 0.433; adjusted P = 0.459)
Improvement in vomiting score: mean difference 0.147 (95% CI −0.108 to 0.401; P = 0.257; adjusted P = 0.265).
These findings suggest that VR intervention was not associated with significant improvements in postoperative symptom recovery compared to the control group (Table 2)."

18-i）仅比较用户的子组分析

电子健康试验中仅比较用户的亚组分析并不罕见，但必须强调这是自我选择的样本，而非随机试验的无偏样本（见16-iii）。

	1	2	3	4	5	
子项一点也不重要	○	○	○	○	○	必不可少

你的纸质上有提到子项18-i吗?

复制粘贴手稿中相关部分（在引号中加上“类似”的引号，以表示直接引用），或者通过提供手稿中未包含的信息来详细说明该项目，或简要说明该项目为何不适用/不相关

您的回答

19）**各组中所有重要的伤害或意外影响**

（具体指导请参见CONSORT有害处）

你的论文是否涉及CONSORT子项19？ *

复制粘贴手稿中相关部分（在引号中加上“类似”的引号，以表示直接引用），或者通过提供手稿中未包含的信息来详细说明该项目，或简要说明该项目为何不适用/不相关

No significant injuries or unexpected impacts occurred in any of the groups.

19-i） 包括隐私泄露、技术问题

包括隐私泄露、技术问题。这不仅包括对参与者的身体“伤害”，还包括被认为或真实的隐私泄露[1]、技术问题以及其他意外/非预期的事件。“非预期影响”也包括非预期的积极影响[2]。

	1	2	3	4	5	
子项一点也不重要	○	○	○	○	○	必不可少

你的纸质上有提到19-i子项吗?

复制粘贴手稿中相关部分（在引号中加上“类似”的引号，以表示直接引用），或者通过提供手稿中未包含的信息来详细说明该项目，或简要说明该项目为何不适用/不相关

您的回答

19-ii） 包含参与者的定性反馈或工作人员/研究人员的观察

如有，包含参与者的定性反馈或工作人员/研究人员对应用优缺点的观察，尤其是指出意外或意外效果或用途的。这包括（如果有的话）人们为何按照开发者意图使用应用的原因。

	1	2	3	4	5	
子项一点也不重要	○	○	○	○	○	必不可少

你的论文是否涉及第19-ii项?

复制粘贴手稿中相关部分（在引号中加上“类似”的引号，以表示直接引用），或者通过提供手稿中未包含的信息来详细说明该项目，或简要说明该项目为何不适用/不相关

您的回答

讨论

22）与结果一致的解释，权衡利弊，并考虑其他相关证据

NPT：此外，还要考虑对照对象的选择、缺乏或部分盲法，以及各组护理提供者或中心的专业能力不均

22-i）重述研究问题并总结数据所建议的答案，从主要结果和过程结果开始（使用）
重新陈述研究问题，并总结数据中提出的答案，从主要结果和过程结果（使用）开始。

	1	2	3	4	5	
子项一点也不重要	○	○	○	○	◉	必不可少

清除所选内容

你的纸质上有提到22-i子项吗？ *
复制粘贴手稿中相关部分（在引号中加上"类似"的引号，以表示直接引用），或者通过提供手稿中未包含的信息来详细说明该项目，或简要说明该项目为何不适用/不相关

"This randomized controlled trial enrolled 131 patients undergoing minimally invasive gynecological surgery to evaluate the efficacy and safety of VR for postoperative pain and anxiety management. The primary outcome—change in VAS score between 6 and 7 hours postoperatively—showed no significant difference between the VR group and the control group (mean difference 0.169, 95% CI −0.271 to 0.608; P =0.449). For secondary outcomes, maximum pain score, anxiety improvement, length of hospital stay, hospitalization cost and incidence of dizziness, nausea and vomiting were also comparable between groups (all P > 0.05). The absence of between-group differences suggests that a single postoperative VR session may provide limited additional benefit beyond routine perioperative care in this population. No increase in adverse events or hospitalization costs was observed in the VR group."

22-ii） 突出未解之新问题，建议未来研究
突出未解之谜，建议未来的研究。

	1	2	3	4	5	
子项一点也不重要	○	○	○	○	○	必不可少

你的纸质上有提到22-ii子项吗？
复制粘贴手稿中相关部分（在引号中加上"类似"的引号，以表示直接引用），或者通过提供手稿中未包含的信息来详细说明该项目，或简要说明该项目为何不适用/不相关

您的回答

20）试验局限性，解决潜在偏差、不精确性及（如相关）分析多重性来源

20-i） 电子健康试验的典型局限性

电子健康试验的典型局限性：电子健康试验参与者很少进行盲法。电子健康试验通常关注多种结局，增加I型错误的风险。讨论因未使用干预/可用性问题、知情同意程序导致的偏见、意外事件等。

	1	2	3	4	5	
子项一点也不重要	○	○	○	○	◉	必不可少

清除所选内容

你的纸质上有提到20-i子项吗？ *

复制粘贴手稿中相关部分（在引号中加上“类似”的引号，以表示直接引用），或者通过提供手稿中未包含的信息来详细说明该项目，或简要说明该项目为何不适用/不相关

"There are also several limitations that need to be addressed in this study. This study was a single-center study and may limit extrapolation of the findings. The study did not collect detailed information about the participants' previous history of mental illness and related medications, leading to potential confounding factors. Furthermore, relatively low postoperative pain and anxiety levels, a short intervention duration, and a modest sample size may have reduced the ability to detect significant differences. Further multicenter studies with longer follow-up and standardized VR protocols are warranted. "

21）试验结果的普遍性（外部效度、适用性）

NPT：根据干预措施、对照对象、患者及参与试验的护理提供者或中心，试验结果的外部效度

21-i）推广到其他群体的可推广性

对其他人群的可推广性：特别讨论对普通互联网人群（非随机对照试验环境）和普通患者群体的普遍性，包括研究结果对其他组织的适用性

	1	2	3	4	5	
子项一点也不重要	○	○	○	○	○	必不可少

你的纸质文件是否涉及21-i子项?

复制粘贴手稿中相关部分（在引号中加上“类似”的引号，以表示直接引用），或者通过提供手稿中未包含的信息来详细说明该项目，或简要说明该项目为何不适用/不相关

您的回答

21-ii）讨论RCT中是否有某些元素在常规应用环境中会有所不同

讨论RCT中是否有某些元素在常规应用环境中会有所不同（例如提示/提醒、更多人类参与、培训课程或其他联合干预），以及如果在RCT外应用干预，这些元素的省略可能对使用、采纳或结果产生什么影响。

	1	2	3	4	5	
子项一点也不重要	○	○	○	○	○	必不可少

你的纸质上有提到第21-ii项吗?

复制粘贴手稿中相关部分（在引号中加上“类似”的引号，以表示直接引用），或者通过提供手稿中未包含的信息来详细说明该项目，或简要说明该项目为何不适用/不相关

您的回答

其他信息

23）注册号及审判登记处名称

你的论文是否涉及CONSORT子项23? *

复制粘贴手稿中相关部分（在引号中加上“类似”的引号，以表示直接引用），或者通过提供手稿中未包含的信息来详细说明该项目，或简要说明该项目为何不适用/不相关

"Chinese Clinical Trial Registry Identifier: ChiCTR2400091244."

24）如果有完整的试验协议，可以访问

你的论文是否涉及CONSORT子项24？ *

引用多媒体附录、其他参考文献，或复制粘贴手稿中相关部分（如“这样”在引号中加引号以表示直接引用），或通过提供手稿中未包含的额外信息来详细说明该项目，或简要说明该项目为何不适用/不相关

The project leader possesses the complete trial protocol.

25）资金来源及其他支持（如药品供应）、资助者的角色

你的论文是否涉及CONSORT子项25？ *

复制粘贴手稿中相关部分（在引号中加上“类似”的引号，以表示直接引用），或者通过提供手稿中未包含的信息来详细说明该项目，或简要说明该项目为何不适用/不相关

"This study was funded by grants from the National Natural Science Foundation of China (NSFC-82301853, to Tengfei Long). ""Tengfei Long was representative of the funder, and participated in designing the study, drafting the manuscript and provided editorial feedback on the final draft."

X27）利益冲突（非CONSORT物品）

X27-i） 说明研究团队与被评估系统的关系

除了通常的利益声明（无论是财务还是其他方面），还应说明研究团队与被评估系统的关系，即说明作者/评估者是否与干预的开发者/赞助者不同或相同。

	1	2	3	4	5	
子项一点也不重要	○	○	○	○	○	必不可少

你的纸质上有提到子项X27-i吗？

复制粘贴手稿中相关部分（在引号中加上"类似"的引号，以表示直接引用），或者通过提供手稿中未包含的信息来详细说明该项目，或简要说明该项目为何不适用/不相关

您的回答

关于CONSORT健康检查清单

使用这份清单后，你对稿件做了修改吗？ *

- ◯ 是的，重大改动
- ◯ 是的，有些小改动
- ◉ 不

使用这份清单后，你做了哪些最重要的改变？

您的回答

你花了多少时间在检查清单上，包括修改手稿？ *

It took about one day to fill out this checklist completely.

使用这份清单后，你认为你的手稿有所提升吗？ *

- ◉ 是的
- ◯ 不
- ◯ 其他:

您想加入CONSORT电子健康集团吗?

例如，参与工作坊并撰写一份“解释与阐述”文档

- ◯ 是的
- ◯ 不
- ◯ 其他:

关于CONSORT EHEALTH还有其他评论或问题吗

您的回答

停止——点击提交前请将此表格保存为PDF。

要生成您填写的记录，建议在提交前先生成本页的PDF（在Mac上，只需选择“打印”，然后选择“打印为PDF”）。

当您向JMIR提交（修订版）论文时，请上传PDF作为补充文件。

不用担心文本框里的某些文字被截断了，因为我们的数据库里仍然保留着完整的信息。谢谢!

最后一步：点击提交!

点击提交，我们的数据库中已有您的答案!

提交

清除表单内容

切勿通过 Google 表单提交密码。

此内容不是由 Google 所创建，Google 不对其作任何担保。 - 服务条款 - 隐私权政策

此表单看起来很可疑吗？ 报告

Google 表单
